# Control of endothelial quiescence by FOXO-regulated metabolites

**DOI:** 10.1038/s41556-021-00637-6

**Published:** 2021-04-01

**Authors:** Jorge Andrade, Chenyue Shi, Ana S. H. Costa, Jeongwoon Choi, Jaeryung Kim, Anuradha Doddaballapur, Toshiya Sugino, Yu Ting Ong, Marco Castro, Barbara Zimmermann, Manuel Kaulich, Stefan Guenther, Kerstin Wilhelm, Yoshiaki Kubota, Thomas Braun, Gou Young Koh, Ana Rita Grosso, Christian Frezza, Michael Potente

**Affiliations:** 1 Angiogenesis and Metabolism Laboratory, Max Planck Institute for Heart and Lung Research, Bad Nauheim, Germany; 2 Medical Research Council Cancer Unit, University of Cambridge, Cambridge, UK; 3 Graduate School of Medical Science and Engineering, Korea Advanced Institute of Science and Technology (KAIST), Daejeon, Korea; 4 Center for Vascular Research, Institute for Basic Science (IBS), Daejeon, Korea; 5 Gene Editing Group, Institute of Biochemistry II, Goethe University, Frankfurt (Main), Germany; 6 Department of Cardiac Development and Remodeling, Max Planck Institute for Heart and Lung Research, Bad Nauheim, Germany; 7 Department of Anatomy, Keio University School of Medicine, Tokyo, Japan; 8 UCIBIO–Unidade de Ciências Biomoleculares Aplicadas, Departamento Ciências da Vida, Faculdade de Ciências e Tecnologia–Universidade Nova de Lisboa Campus de Caparica, Caparica, Portugal; 9 Instituto de Medicina Molecular, Faculdade de Medicina, Universidade de Lisboa, Lisboa, Portugal; 10 Berlin Institute of Health (BIH) at Charité–Universitätsmedizin Berlin, Berlin, Germany; 11 Max Delbrück Center for Molecular Medicine (MDC), Berlin, Germany; 12 Present Address: Cold Spring Harbor Laboratory, Cold Spring Harbor, NY USA; 13 Present Address: Department of Oncology and Ludwig Institute for Cancer Research, University of Lausanne and Centre Hospitalier Universitaire Vaudois, Epalinges, Switzerland

**Keywords:** Angiogenesis, Angiogenesis, Mitochondria

## Abstract

Endothelial cells (ECs) adapt their metabolism to enable the growth of new blood vessels, but little is known how ECs regulate metabolism to adopt a quiescent state. Here, we show that the metabolite *S*-2-hydroxyglutarate (*S*-2HG) plays a crucial role in the regulation of endothelial quiescence. We find that *S*-2HG is produced in ECs after activation of the transcription factor forkhead box O1 (FOXO1), where it limits cell cycle progression, metabolic activity and vascular expansion. FOXO1 stimulates *S*-2HG production by inhibiting the mitochondrial enzyme 2-oxoglutarate dehydrogenase. This inhibition relies on branched-chain amino acid catabolites such as 3-methyl-2-oxovalerate, which increase in ECs with activated FOXO1. Treatment of ECs with 3-methyl-2-oxovalerate elicits *S*-2HG production and suppresses proliferation, causing vascular rarefaction in mice. Our findings identify a metabolic programme that promotes the acquisition of a quiescent endothelial state and highlight the role of metabolites as signalling molecules in the endothelium.

## Abstract (web summary)

Andrade et al. show that FOXO1 regulates mitochondrial metabolism to stimulate the production of the metabolite *S*-2HG to promote acquisition of a quiescent endothelial state.

## Main

Homeostasis of the blood vasculature relies on a single layer of endothelial cells (ECs) forming the inner surface of all vessels. In most adult tissues, ECs reside in a non-cycling, quiescent state, which is critical for their function as a barrier and signalling interface^1,2^. This resting state is reversible, and ECs can (re-)activate, enter the cell cycle and expand to meet physiological or stress-induced demands. While the processes of endothelial activation and proliferation are being defined with increasing molecular resolution, the mechanisms leading to the acquisition of a quiescent phenotype remain poorly understood.

Previous studies have demonstrated that the forkhead box O (FOXO) transcription factor FOXO1 is a critical driver of endothelial quiescence^3,4^. FOXO1 activity is controlled by the phosphatidylinositol-3-OH kinase–AKT pathway that inhibits FOXO1 transcriptional responses through AKT-mediated phosphorylation^5,6^. ECs are highly sensitive to changes in FOXO1 activity, as both EC-specific deletion and forced activation are early embryonic lethal in mice^3,4,7,8^. Loss of FOXO1 leads to unregulated endothelial proliferation and vascular overgrowth, whereas forced activation causes premature quiescence and vascular rarefaction. FOXO1 promotes quiescence, in part, by suppressing MYC signalling, which leads to a reduction in endothelial metabolic activity^4^. Yet, the FOXO1-regulated metabolic programmes in ECs are largely unknown. Given the importance of endothelial metabolic regulation for vascular growth and function^9–19^ and the sensitivity of ECs towards changes in FOXO1 activity, we sought to investigate the global metabolic profile of FOXO1-induced ECs.

## Results

### FOXO1 activation induces 2-hydroxyglutarate generation in ECs

**Extended Data Fig. 1:**
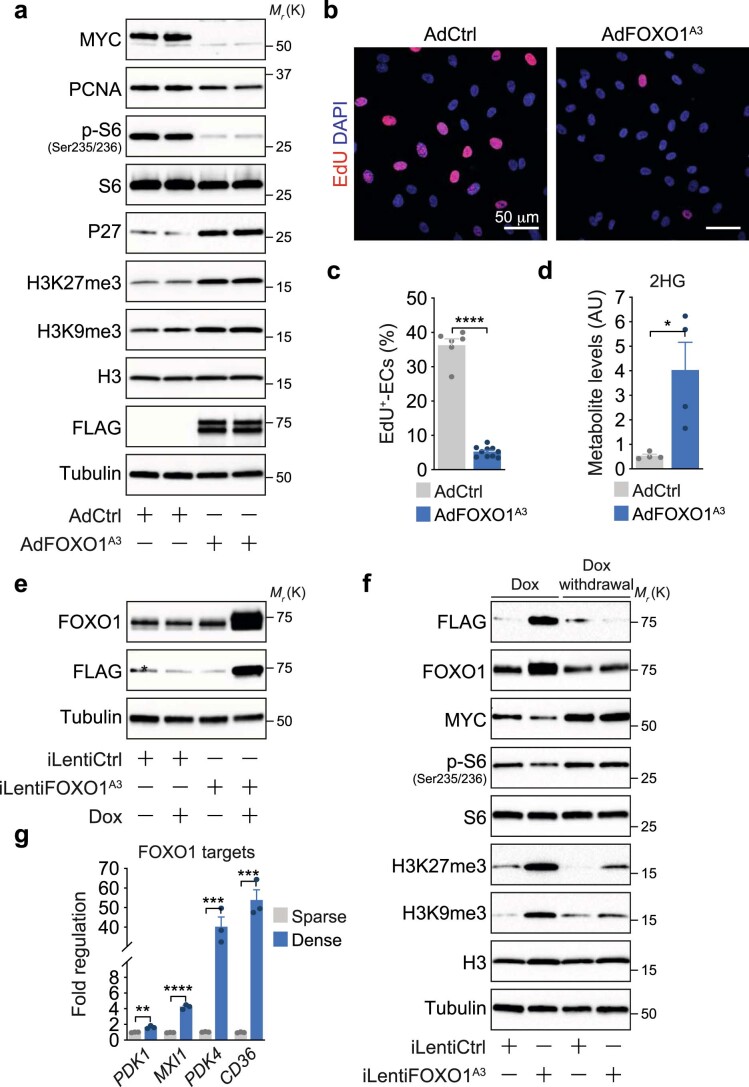
Foxo1 promotes a quiescent endothelial phenotype. **a**, Immunoblot analysis of quiescence-associated protein markers in HUVECs transduced with a FOXO1^A3^ (AdFOXO1^A3^) or control (AdCtrl) adenovirus. A FLAG antibody was used to validate the expression of the FLAG-tagged FOXO1^A3^ mutant. Tubulin served as loading control. **b**, Confocal images showing decreased EdU-incorporation in HUVECs transduced with AdFOXO1^A3^. The analysis was performed 24h after transduction. DAPI was used to identify endothelial nuclei. **c**, Quantification of EdU-incorporation in AdCtrl and FOXO1^A3^-expressing HUVECs. Values represent the percentage of EdU-labelled ECs, (n=6, 10 independent samples for AdCtrl and AdFOXO1^A3^). **d**, 2HG levels in AdCtrl- and AdFOXO1^A3^-transduced HUVECs measured by LC-MS, (n=4 independent samples). AU, arbitrary units. **e**, Immunoblot analysis of HUVECs transduced with a doxycycline-inducible control- (iLentiCtrl) or FOXO1^A3^-encoding lentivirus (iLentiFOXO1^A3^) showing expression of the FLAG-tagged FOXO1^A3^ mutant after doxycycline (Dox) treatment for 48h. The asterisk (*) denotes an unspecific protein detected by the FLAG antibody. **f**, Immunoblot analysis showing that the FOXO1-induced quiescence signature is reversible. HUVECs transduced with iLentiCtrl or iLentiFOXO1^A3^ were treated with Dox for 48h, after which Dox was removed from the culture media. HUVECs were then cultured for additional 48h. **g**, Quantitative RT-PCR (RT-qPCR) showing increased expression of canonical FOXO1 target genes in dense or sparse HUVEC cultures. Values are normalized to β-actin and represent fold-change regulation relative to control, (n=3 independent samples). Western blot data in **a**, **e** and **f** were from the respective experiment, processed in parallel, and are representative of at least three independent experiments. **c**, **d** and **g**, Data represent mean ± s.e.m.; a two-tailed unpaired *t*-test was used; *P<0.05; **P<0.01; ***P<0.001; ****P<0.0001. The numerical data, unprocessed western blots and *P* values are provided as source data. Source data

We performed untargeted metabolomics of human umbilical vein endothelial cells (HUVECs) that were transduced with adenoviruses encoding a constitutively active FOXO1 mutant (AdFOXO1^A3^) or green fluorescent protein (GFP) as a control (AdCtrl). This mutant has the three AKT phosphorylation sites replaced by alanine (→FOXO1^A3^), which renders FOXO1 in the nucleus^5,6^. ECs expressing FOXO1^A3^ were in a reversible proliferation arrest and exhibited a molecular signature characteristic of cellular quiescence, including suppression of the proliferation markers MYC and proliferating cell nuclear antigen (PCNA), as well as induction of the cell cycle inhibitor p27 (CDKN1B) and the repressive histone mark histone H3 lysine 27 trimethylation (H3K27me3)^20–23^ (Extended Data Fig. 1a–c,f).

**Fig. 1:**
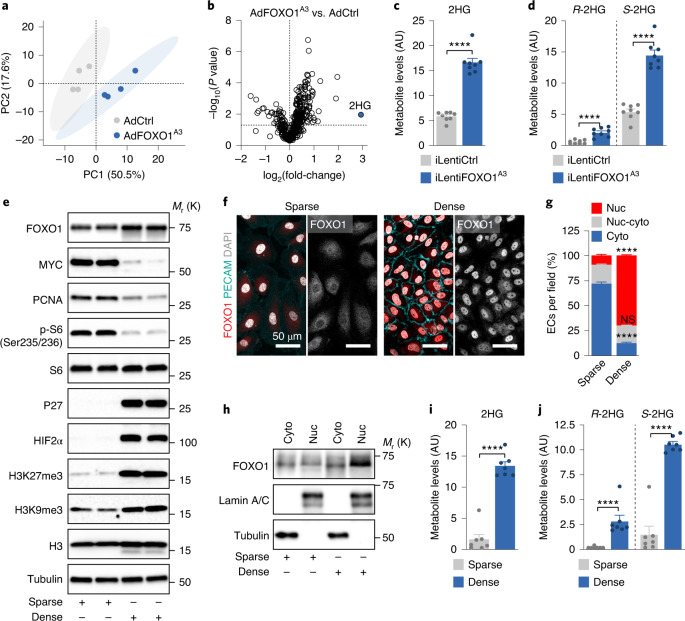
Activation of FOXO1 signalling induces the generation of *S*-2HG in ECs. **a**, PCA score plot showing distinct metabolic signatures in control (AdCtrl) and FOXO1^A3^-expressing (AdFOXO1^A3^) HUVECs (*n* = 4 independent samples). The percentage variance explained by each principal component (PC) is shown in parentheses. **b**, Volcano plot of metabolites showing 2HG as the most increased metabolite in FOXO1^A3^-expressing HUVECs. **c**, 2HG metabolite levels measured by LC–MS in HUVECs transduced with a doxycycline-inducible control-encoding (iLentiCtrl) or FOXO1^A3^-encoding (iLentiFOXO1^A3^) lentivirus (*n* = 8 independent samples). AU, arbitrary units. **d**, Chiral derivatization and enantioselective LC–MS measurement of *R*- and *S*-2HG levels in HUVECs transduced with iLentiCtrl or iLentiFOXO1^A3^ (*n* = 8 independent samples). **e**, Immunoblot analysis of HUVECs cultured in sparse and dense conditions. HUVECs in dense (contact-inhibited) conditions express markers linked to cellular quiescence. K, 1,000. **f**,**g**, Immunofluorescence analysis (**f**) and quantification (**g**) of the subcellular localization of FOXO1 (red) in HUVECs cultured in sparse and dense conditions. The isolated FOXO1 signal is shown on the right side of each image in grey (*n* = 14 (sparse condition) or 8 (dense condition) independent samples). DAPI (grey), nuclei; PECAM (cyan), intercellular endothelial junctions. Cyto, cytoplasmic; Nuc, nuclear; Nuc–cyto, nuclear–cytoplasmic. **h**, FOXO1 protein levels in cytoplasmic and nuclear fractions isolated from HUVECs cultured under sparse and dense conditions. Lamin A/C and tubulin were used as nuclear and cytoplasmic markers, respectively. **i**, 2HG metabolite levels in HUVECs cultured in sparse (FOXO1 inactive) and dense (FOXO1 active) culture conditions (*n* = 7 independent samples). **j**, Enantioselective LC–MS measurement of *R*- and *S*-2HG in sparse and dense HUVEC cultures (*n* = 7 independent samples). Western blot data in **e** and **h** are from the respective experiment, processed in parallel, and are representative of at least three independent experiments. For **c**, **d**, **g**, **i** and **j**, data represent the mean ± s.e.m.; two-tailed unpaired *t*-test, ****P < 0.0001, NS, not significant. The numerical data, unprocessed western blots and *P* values are provided as source data. Source data

Principal component analysis (PCA) of the intracellular metabolites collected 24 h post-transduction revealed a separate clustering of control and FOXO1^A3^-transduced ECs (Fig. 1a). ECs with nuclear FOXO1 exhibited increased levels of several branched-chain amino acid (BCAA) metabolites and of early tricarboxylic acid (TCA) cycle intermediates (Supplementary Table 1). Notably, the most increased metabolite (7.6-fold) was 2-hydroxyglutarate (2HG) (Fig. 1b and Extended Data Fig. 1d), which is a chiral molecule derived from 2-oxoglutarate (2OG) that functions as a competitive inhibitor of many 2OG-dependent dioxygenases. Among these are Jumonji C (JmjC) domain-containing lysine demethylases and hypoxia-inducible factor (HIF)-regulating prolyl hydroxylases (PHDs)^24–29^, which suggests that FOXO1 may exert profound effects on gene expression via regulation of this metabolite.

We therefore confirmed the regulation of 2HG using a lentiviral system for doxycycline-inducible expression of FOXO1^A3^ (Fig. 1c and Extended Data Fig. 1e) and contact inhibition of endothelial proliferation—a stimulus for nuclear translocation and activation of endogenous FOXO1 (Fig. 1e–i and Extended Data Fig. 1g). Indeed, we observed an 8.4-fold increase in 2HG abundance in dense (contact-inhibited) culture conditions, whereby FOXO1 is an induced and predominantly nuclear protein and in which canonical FOXO1 target genes are highly transcribed (for example, MAX interactor 1 (*MXI1*), pyruvate dehydrogenase kinase 1 (*PDK1*), pyruvate dehydrogenase kinase 4 (*PDK4*) and CD36 molecule (*CD36*)) (Fig. 1f–i and Extended Data Fig. 1g). Thus, ECs generate 2HG when FOXO1 signalling is activated.

### FOXO1-activated ECs produce *S*-2HG, which inhibits 2OG-dependent dioxygenases

**Extended Data Fig. 2:**
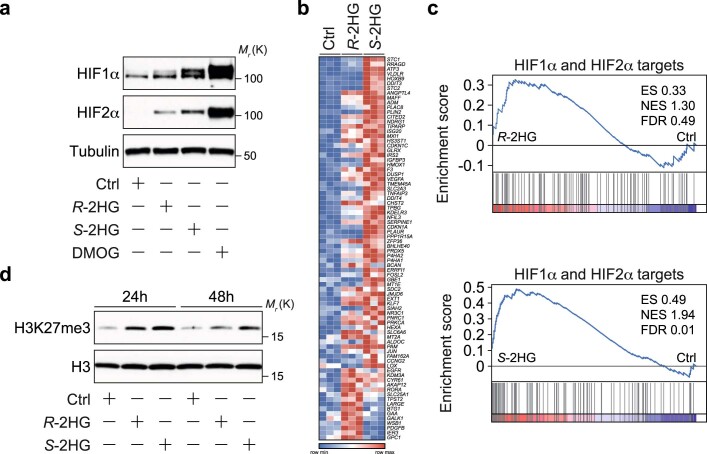
*R*- and *S*-2HG reduce the activity of 2-oxoglutarate-dependent dioxygenases in ECs. **a**, Immunoblot analysis of HIF1α and HIF2α protein abundance in HUVECs stimulated with cell-permeable *R*- or *S*-2HG for 24h. HUVECs treated with vehicle (DMSO) were used as a control (Ctrl). The PHD inhibitor DMOG, which stabilizes HIF protein levels, was used as a positive control. **b**, Heatmap of hypoxia associated genes that are differentially regulated in control (Ctrl) versus *R*-2HG or *S*-2HG-treated HUVECs. DMSO was used as a vehicle control. Cells were stimulated for 24h before total mRNA was collected for RNA-seq analysis, (n=3 independent samples). **c**, Gene set enrichment analysis (GSEA) showing a HIF gene expression signature in HUVECs treated with *R*- and *S*-2HG for 24h when compared to Ctrl. ES, enrichment score; NES, normalized enrichment score; FDR, false discovery rate. **d**, Immunoblot analysis showing increased histone H3 lysine 27 tri-methylation (K27me3) levels in HUVECs treated with *R*-2HG or *S*-2HG when compared to Ctrl. Cells were analysed 24 or 48h after stimulation. Total levels of histone H3 are shown as protein loading control. Western blot data in **a** and **d** were from the respective experiment, processed in parallel, and are representative of at least three independent experiments. Unprocessed western blots are provided as source data. Source data

Two conformations of 2HG exist, the *R*-2HG and *S*-2HG enantiomers, which have similar but not identical functions^30–32^. Chiral derivatization followed by liquid chromatography–mass spectrometry (LC–MS) revealed that FOXO1 induces the preferential generation of *S*-2HG, which is also the more abundant enantiomer in ECs (Fig. 1d,j). To explore whether 2HG regulates the activity of endothelial 2OG-dependent dioxygenases, we first analysed HIFs, which are marked by PHDs for proteasomal degradation under normoxic conditions. In line with an inhibitory effect of 2HG on PHDs^30,31,33–35^, incubation of HUVECs with cell-permeable forms of *R*-2HG or *S*-2HG increased HIF1α and HIF2α protein abundance and enhanced HIF target gene expression (Extended Data Fig. 2a–c). Interestingly, *S*-2HG had more profound effects on HIF responses than *R*-2HG (Extended Data Fig. 2a–c). Similar results were obtained for H3K27me3, which is targeted by the 2OG-dependent JmjC histone demethylases (Extended Data Fig. 2d), which suggests that *S*-2HG is the more potent inhibitor of 2OG-dependent dioxygenases in ECs. Therefore, we focused our further analysis on this enantiomer.

### *S*-2HG promotes a quiescent endothelial state

**Fig. 2:**
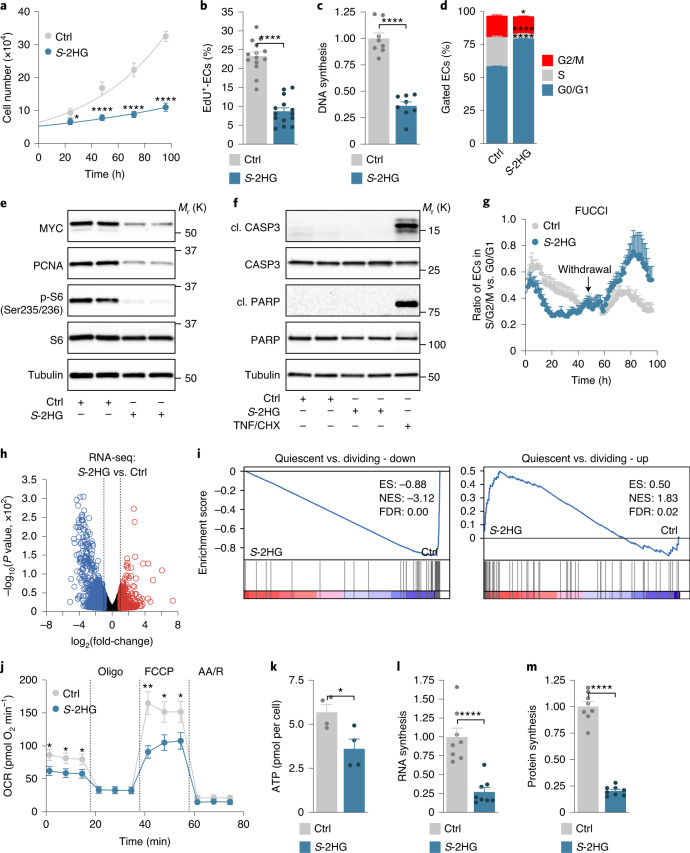
*S*-2HG supports a quiescent endothelial phenotype. **a**, Growth curves of HUVECs stimulated with DMSO (Ctrl) or cell-permeable *S*-2HG (*n* = 16 independent samples). **b**, EdU incorporation in control or *S*-2HG-treated HUVECs at 48 h. The percentage of EdU-positive ECs is shown (*n* = 14 independent samples). **c**, DNA synthesis is reduced in HUVECs treated with *S*-2HG for 48 h. Values represent the fold-change relative to DMSO-treated HUVECs (*n* = 8 independent samples). **d**, Cell-cycle analysis of control and *S*-2HG-stimulated HUVECs 48 h after treatment (*n* = 4 independent samples). **e**, Immunoblotting of proliferation and growth-associated proteins in HUVECs treated with *S*-2HG for 24 h. **f**, Immunoblot analysis of the apoptotic markers cleaved (cl.) caspase-3 (CASP3) and cleaved PARP showing that *S*-2HG does not cause cell death in HUVECs. TNFα (TNF) and cycloheximide (CHX) co-stimulation was used as a positive control. **g**, Time-course analysis of HUVECs transduced with the dual FUCCI reporter. ECs were treated with vehicle or *S*-2HG for 48 h, followed by withdrawal of the treatment and further analysis for 48 h (*n* = 3 independent samples). **h**, Volcano plot of differentially expressed genes in control or *S*-2HG-treated HUVECs at 24 h. Genes with a *P* value cut-off of ≤0.05 and a fold-change ≥ or ≤2 are shown (*n* = 3 independent samples). **i**, GSEA plots of quiescent versus dividing-down and quiescent versus dividing-up gene sets in the transcriptomes of control or *S*-2HG-treated HUVECs. ES, enrichment score; FDR, false discovery rate; NES, normalized enrichment score. **j**, OCRs in control or *S*-2HG-treated HUVECs (*n* = 9 independent samples). AA/R, antimycin A/rotenone; Oligo, oligomycin. **k**, ATP levels in HUVECs 48 h after treatment with vehicle or *S*-2HG (*n* = 4 independent samples). **l**, RNA synthesis is decreased, as measured by ^14^C-glucose incorporation into RNA, in HUVECs treated with *S*-2HG for 48 h. Values are represented as the fold-change relative to control (*n* = 8 independent samples). **m**, Protein synthesis is decreased, as measured by ^3^H-tyrosine incorporation into protein, in HUVECs treated with *S*-2HG for 48 h. Values are represented as the fold-change relative to control (*n* = 8 independent samples). Western blot data in **e** and **f** are from the respective experiment, processed in parallel, and are representative of at least three independent experiments. For **a**–**d**, **g** and **j**–**m**, the data represent the mean ± s.e.m.; two-tailed unpaired *t*-test, *P < 0.05, **P < 0.01, ****P < 0.0001. The numerical data, unprocessed western blots and *P* values are provided as source data. Source data

**Extended Data Fig. 3:**
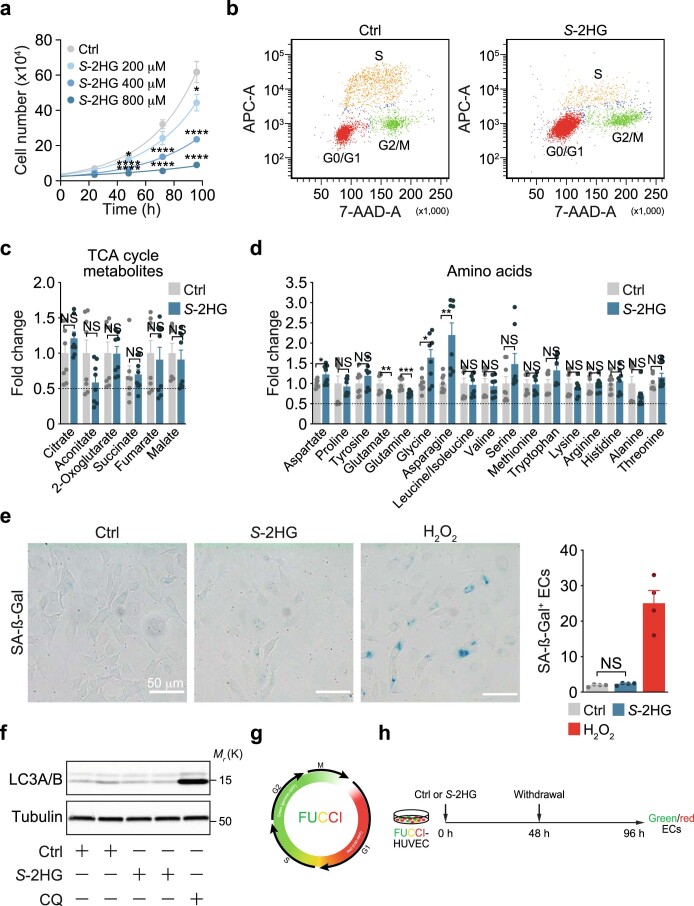
*S*-2HG is a cell cycle-arresting metabolite. **a**, Proliferation curves comparing HUVECs treated with DMSO (Ctrl) or cell-permeable *S*-2HG, showing a dose-dependent reduction in *S*-2HG-stimulated HUVECs at the indicated concentrations and time points, (n=10 independent samples). **b**, Representative flow cytometry density plots showing the cell cycle phase distribution in HUVECs treated with vehicle (Ctrl) or *S*-2HG for 48 hours. **c**, Relative abundance of TCA cycle metabolites in Ctrl and *S*-2HG-treated HUVECs. LC-MS measurements were performed 48h after stimulation, (n=8 independent samples). **d**, Relative abundance of amino acids in Ctrl and *S*-2HG-treated HUVECs. LC-MS measurements were performed 48h after stimulation, (n=8 independent samples). **e**, Representative images and quantification of senescence-associated β-galactosidase (SA-β-Gal) staining showing that S-2HG does not induce cellular senescence. HUVECs were stimulated with DMSO (Ctrl) or *S*-2HG for 48h. Hydrogen peroxide (H_2_O_2_) stimulated cells were used as a positive control, (n=4 independent samples). **f**, LC3A/B immunoblot analysis showing that *S*-2HG treatment for 48h does not induce autophagy in ECs. Chloroquine (CQ) treated HUVECs were used as a positive control. **g**, Visualization of cell cycle progression using the dual fluorescence ubiquitination-based cell cycle indicator (FUCCI) reporter. Cells with red-labelled nuclei (expressing mCherry-hCdt1(30/120)) are in G0/G1 while cells with green labelled nuclei (expressing mVenus-hGeminin(1/110)) are in the S/G2/M cell cycle phase. Yellow nuclei indicate temporal co-expression of both reporters. **h**, Experimental timeline for the cell cycle analysis in HUVECs transduced with the FUCCI reporter. The ratio between green and red cells indicates the percentage of ECs in S/G2/M compared to G0/G1, respectively. Western blot data in **f** were from the respective experiment, processed in parallel, and are representative of at least three independent experiments. **a** and **c-e**, Data represent mean ± s.e.m.; a two-tailed unpaired *t*-test was used; *P<0.05; ** *P*<0.01; ***P<0.001; **** *P*<0.0001; NS, not significant. The numerical data, unprocessed western blots and *P* values are provided as source data. Source data

To characterize the role of *S*-2HG in ECs, we incubated HUVECs with different *S*-2HG concentrations and noted a time- and dose-dependent suppression of endothelial proliferation (Fig. 2a–c and Extended Data Fig. 3a). *S*-2HG-treated ECs were arrested in the G0/G1 phase of the cell cycle, and markers of cell growth and proliferation, including MYC, PCNA and phosphorylated ribosomal protein S6, were diminished (Fig. 2d,e and Extended Data Fig. 3b). *S*-2HG did not cause metabolic distress (depletion of a particular metabolite), senescence or apoptotic cell death, as reporters of these processes were unaltered or not detectable (Fig. 2f and Extended Data Fig. 3c–f). To further characterize this antiproliferative effect, we transduced HUVECs with a lentiviral vector encoding the fluorescent ubiquitination-based cell cycle indicator (FUCCI). This dual-colour reporter labels nuclei of cells in the G0/G1 phase in red and those in the S/G2/M phases in green (Extended Data Fig. 3g,h). *S*-2HG caused a progressive increase in ECs with red fluorescence, thereby confirming the G0/G1 arrest (Fig. 2g and Supplementary Videos 1 and 2). However, *S*-2HG withdrawal led to the reappearance of ECs with green fluorescent nuclei (Fig. 2g and Supplementary Videos 1 and 2), which indicates that the *S*-2HG-induced proliferation arrest is reversible.

**Extended Data Fig. 4:**
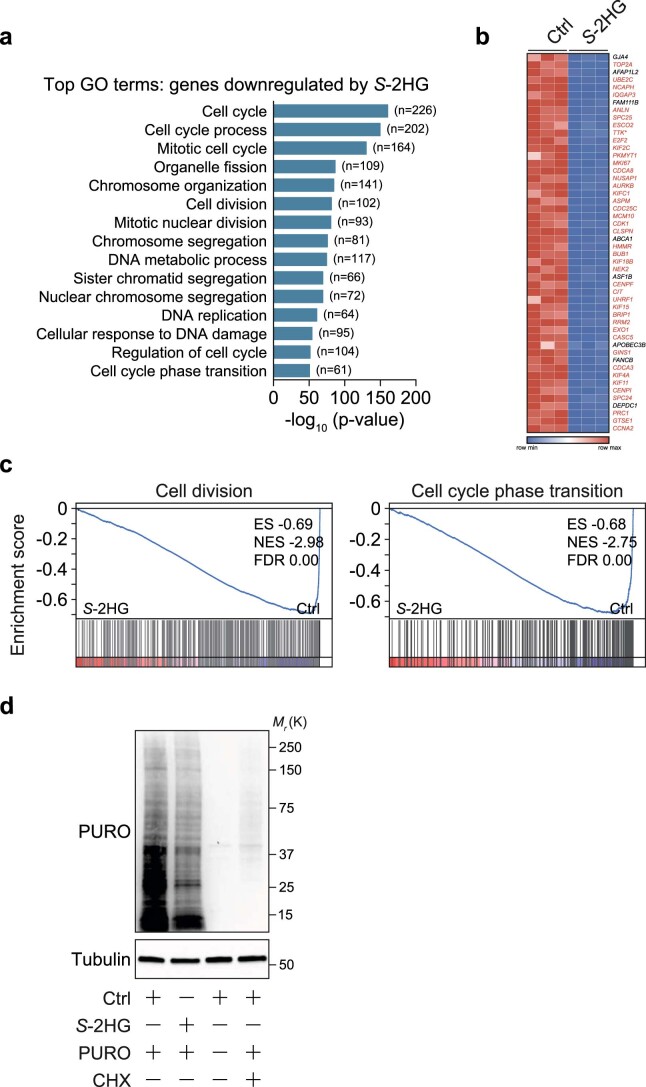
*S*-2HG promotes a quiescent state in ECs. **a**, Gene ontology (GO) analysis showing top GO terms of genes that are downregulated in *S*-2HG-treated HUVECs as determined by RNA-seq analysis at 24h post treatment. DMSO-treated HUVECs were used as a control (Ctrl), (n=3 independent samples). **b**, Heatmap showing the top down-regulated genes in the transcriptome of HUVECs treated with *S*-2HG for 24h. Transcripts highlighted in red are cell-cycle and proliferation related genes. **c**, GSEA plots of cell division and cell cycle phase transition gene sets in the transcriptomes of HUVECs treated with *S*-2HG or solvent (Ctrl) for 24h. ES, enrichment score; NES, normalized enrichment score; FDR, false discovery rate. **d**, *S*-2HG decreases endothelial protein synthesis. Immunoblot analyses showing reduced incorporation of puromycin (PURO) into nascent polypeptide chains in whole-cell lysates of HUVECs treated with *S*-2HG or solvent (Ctrl) for 48h. Cycloheximide (CHX) stimulation was used as a positive control to block protein synthesis. Western blot data were from the respective experiment, processed in parallel, and are representative of at least three independent experiments. Unprocessed western blots are provided as source data. Source data

RNA sequencing (RNA-seq) of HUVECs treated with vehicle or *S*-2HG for 24 h revealed that *S*-2HG regulates genes strongly linked to proliferation (Fig. 2h, Extended Data Fig. 4a–c and Supplementary Table 2). A total of 43 out of the 50 most downregulated genes are involved in cell cycle progression and cell division, including DNA topoisomerase II alpha (*TOP2A*), anillin (*ANLN*), marker of proliferation Ki-67 (*MKI67*) and cyclin-dependent kinase 1 (*CDK1*) (Extended Data Fig. 4b). Gene set enrichment analysis (GSEA) corroborated this notion (Fig. 2i and Extended Data Fig. 4c). Moreover, numerous quiescence-associated genes^36^, including those that are not directly linked to cell proliferation, were enriched in *S*-2HG-treated ECs (Fig. 2i). These data suggest that *S*-2HG regulates not only endothelial proliferation but also, more generally, promotes a quiescent endothelial state. Consistent with this idea, *S*-2HG-treated ECs showed a number of cellular phenotypes that are characteristic for quiescent cells^23^, including lower metabolic activity and reduced RNA and protein synthesis (Fig. 2j–m and Extended Data Fig. 4d).

### *S*-2HG limits the angiogenic behaviour of ECs

**Fig. 3:**
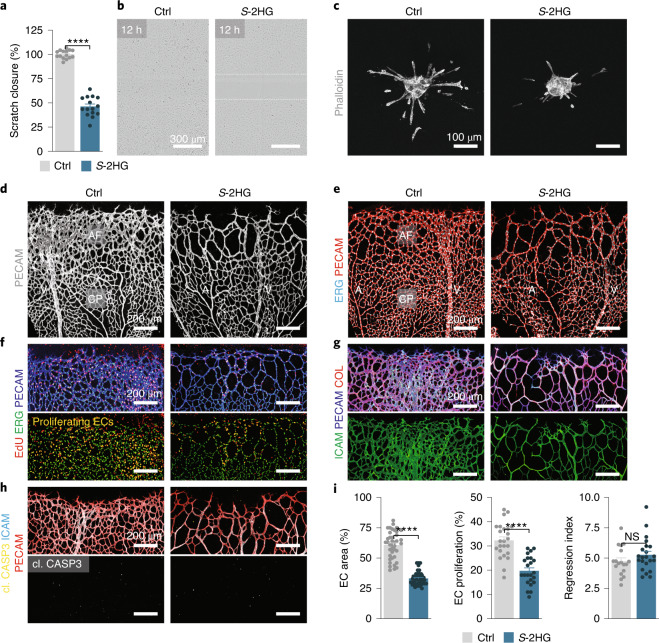
*S*-2HG restrains the angiogenic activity of ECs. **a**,**b**, *S*-2HG reduces the motile behaviour of cultured HUVECs in a scratch-wound assay. Quantification (**a**) and representative bright-field images (**b**) from control and *S*-2HG-treated HUVECs (*n* = 15 independent samples). **c**, Confocal images of phalloidin-labelled (grey) HUVEC spheroids showing reduced endothelial sprouting in *S*-2HG treated spheroids. Images were taken 24 h after treatment. **d**, PECAM1 (PECAM) immunofluorescence staining (grey) in P7 mouse retinas showing decreased vascular density after a single intravitreal injection of cell-permeable *S*-2HG at P5. Controls were obtained by injection of DMSO (vehicle, Ctrl) in the contralateral eye. A, artery; AF, angiogenic front; CP, capillary plexus; V, vein. **e**, Confocal images of P7 retinas from control and *S*-2HG-injected mice stained for ERG (cyan) and PECAM (red). **f**, Immunofluorescence staining for EdU (red), ERG (green) and PECAM (blue) in P7 mouse retinas of control and *S*-2HG mice. Proliferating (EdU and ERG double-positive) ECs are shown in yellow. **g**, Confocal images of retinas from control and *S*-2HG-injected mice stained for ICAM2 (ICAM, green), PECAM (blue) and collagen IV (COL, red). **h**, Immunofluorescence images of cleaved caspase-3 (yellow), ICAM (cyan) and PECAM (red) of P7 retinas from control and *S*-2HG-injected mice, excluding excessive apoptotic cell death in *S*-2HG-treated mice. Note that most of the cleaved caspase-3 signals come from nonvascular (ICAM/PECAM-negative) cells. **i**, Quantification of angiogenic parameters in retinas of control and *S*-2HG-injected P7 mice, as indicated (EC area: *n* = 21 samples each for control and *S*-2HG; EC proliferation: *n* = 24 each samples for control and *S*-2HG; regression index: *n* = 18 and 22 samples for control and *S*-2HG, respectively). For **a** and **i**, the data represent the mean ± s.e.m.; two-tailed unpaired *t*-test, ****P < 0.0001. The numerical data and *P* values are provided as source data. Source data

**Extended Data Fig. 5:**
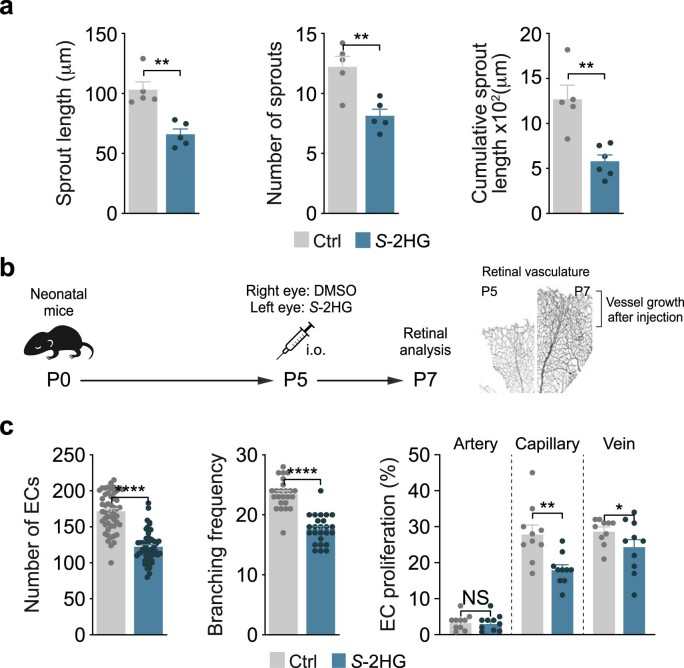
*S*-2HG limits the angiogenic capacity of ECs. **a**, Quantifications of vascular parameters showing reduced endothelial sprouting capacity in *S*-2HG treated HUVEC spheroids, (n=5 independent samples). **b**, Experimental timeline for the retinal analysis after intraocular injection of a single dose of vehicle (DMSO, Ctrl) or *S*-2HG. The images of PECAM-labelled retinas on the right illustrate the extent of angiogenic growth between P5 and P7 in wild-type mice. **c**, Quantifications of vascular parameters in P7 Ctrl and *S*-2HG injected mouse retinas as indicated, (Number ECs: n=56, 51 samples for Ctrl and *S*-2HG; Branching frequency: n=24, 25 samples for Ctrl and *S*-2HG; EC proliferation for Artery, Capillary and Vein: n=10, 10 samples for Ctrl and *S*-2HG). **a** and **c**, Data represent mean ± s.e.m.; a two-tailed unpaired *t*-test was used; *P<0.05; ** *P*<0.01; **** *P*<0.0001; NS, not significant. The numerical data and *P* values are provided as source data. Source data

To seek further evidence of a quiescence-promoting function of *S*-2HG, we visualized endothelial angiogenic behaviour in an in vitro scratch assay and found that *S*-2HG-treated ECs were less motile (Fig. 3a,b and Supplementary Videos 3 and 4). Moreover, analysis of three-dimensional endothelial spheroid cultures revealed that *S*-2HG-treated spheroids formed fewer and shorter sprouts compared to controls (Fig. 3c and Extended Data Fig. 5a). We validated the relevance of these findings by studying blood vessel growth in the mouse retina, in which blood vessels develop postnatally in a highly stereotypical manner. Postnatal day 5 (P5) pups received a single intraocular injection of *S*-2HG in one eye and vehicle in the other (Extended Data Fig. 5b). Analysis of the retinal vasculature 2 days later (at P7) showed a poorly connected and hypocellular endothelial network in *S*-2HG-treated retinas (Fig. 3d,e,i and Extended Data Fig. 5c). This phenotype was particularly evident at the angiogenic front, where most of the endothelial growth and proliferation occurs, while central parts of the retinal vasculature that formed before the injection were less affected (Fig. 3d,e). The sparse network was not due to increased vessel pruning or apoptotic cell death, since analysis of endothelial-less basement membrane sleeves or cleaved caspase-3-positive ECs did not reveal significant changes (Fig. 3g–i). Instead, we found a marked suppression of EC proliferation, which was indicated by a reduction in 5-ethynyl-2′-deoxyuridine (EdU)-positive endothelial nuclei (Fig. 3f,i and Extended Data Fig. 5c). These data are consistent with an anti-angiogenic activity of *S*-2HG in ECs and suggest that *S*-2HG enforces the pro-quiescent function of FOXO1.

### FOXO1 induces *S*-2HG generation by inhibiting the OGDH enzyme

**Fig. 4:**
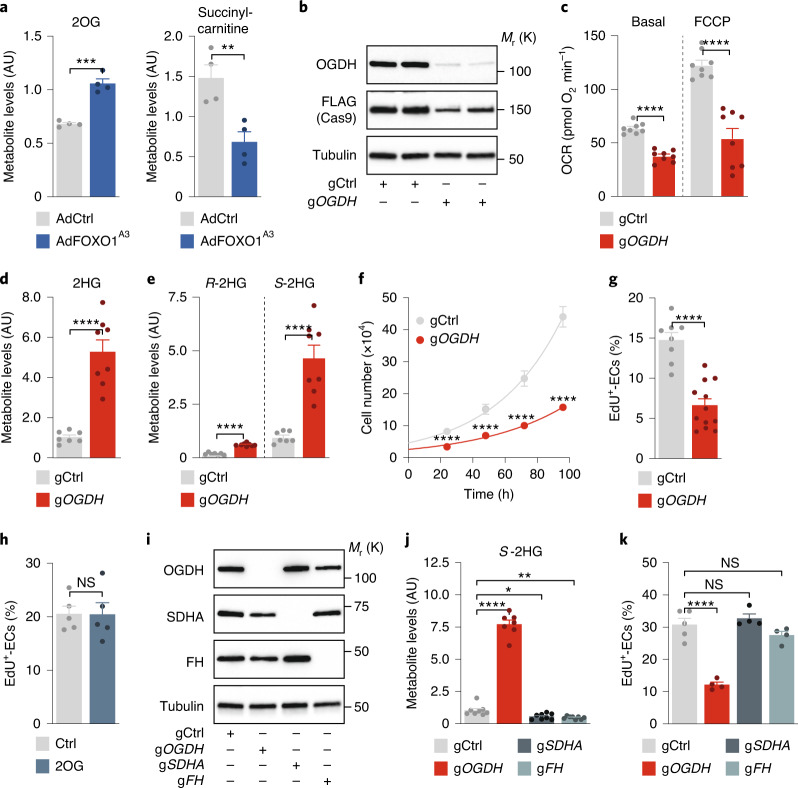
OGDH inactivation induces *S*-2HG generation and limits endothelial proliferation. **a**, Metabolite levels of 2OG and succinyl-carnitine (a surrogate for succinyl-CoA) in HUVECs transduced with control (AdCtrl) or FOXO1^A3^-encoding (AdFOXO1^A3^) adenoviruses (*n* = 4 independent samples). **b**, Immunoblot analysis of OGDH protein abundance in control and OGDH-depleted HUVECs. Cells were generated by lentiviral transduction of FLAG-tagged Cas9 nuclease and control (gCtrl) or OGDH-targeting (g*OGDH*) gRNAs. **c**, OCRs in gCtrl- and g*OGDH*-transduced HUVECs under basal conditions and in response to FCCP (*n* = 8 independent samples). **d**, 2HG metabolite levels in gCtrl and g*OGDH* HUVECs measured by LC–MS (*n* = 7 (gCtrl) and 8 (g*OGDH*) independent samples). **e**, Enantioselective LC–MS measurement of *R*- and *S*-2HG levels in gCtrl and g*OGDH* HUVECs (*n* = 7 (gCtrl) and 8 (g*OGDH*) independent samples). **f**, Cell-proliferation curves comparing gCtrl and g*OGDH* HUVECs (*n* = 12 independent samples). **g**, EdU incorporation in control and OGDH-deficient HUVECs. The percentage of EdU-positive ECs is shown (*n* = 8 (gCtrl) and 12 (g*OGDH*) independent samples). **h**, EdU incorporation in control and cell-permeable 2OG-treated HUVECs. The percentage of EdU-positive ECs is shown (*n* = 5 independent samples). **i**, Immunoblot analysis of OGDH, SDHA and FH protein levels in control (gCtrl) and CRISPR–Cas9-engineered HUVECs (g*OGDH*, g*SDHA* or g*FH*). **j**, LC–MS measurement of 2HG enantiomers showing that OGDH-deficient HUVECs (g*OGDH*), but not SDHA- or FH-deficient cells, have increased *S*-2HG levels (*n* = 8, 7, 8 and 8 independent samples for gCtrl, g*OGDH*, g*SDHA* and g*FH*, respectively). **k**, EdU incorporation in gCtrl, g*OGDH*, *gSDHA* and *gFH* HUVECs. The percentage of EdU-positive ECs is shown (*n* = 5, 4, 4 and 4 independent samples for gCtrl, g*OGDH*, g*SDHA* and g*FH*, respectively). Western blot data in **b** and **i** are from the respective experiment, processed in parallel, and are representative of at least three independent experiments. For **a**, **c**, **d**–**h**, **j** and **k**, the data represent the mean ± s.e.m.; two-tailed unpaired *t*-test, *P < 0.05, **P < 0.01, ***P < 0.001, ****P < 0.0001, NS, not significant. The numerical data, unprocessed western blots and *P* values are provided as source data. Source data

**Extended Data Fig. 6:**
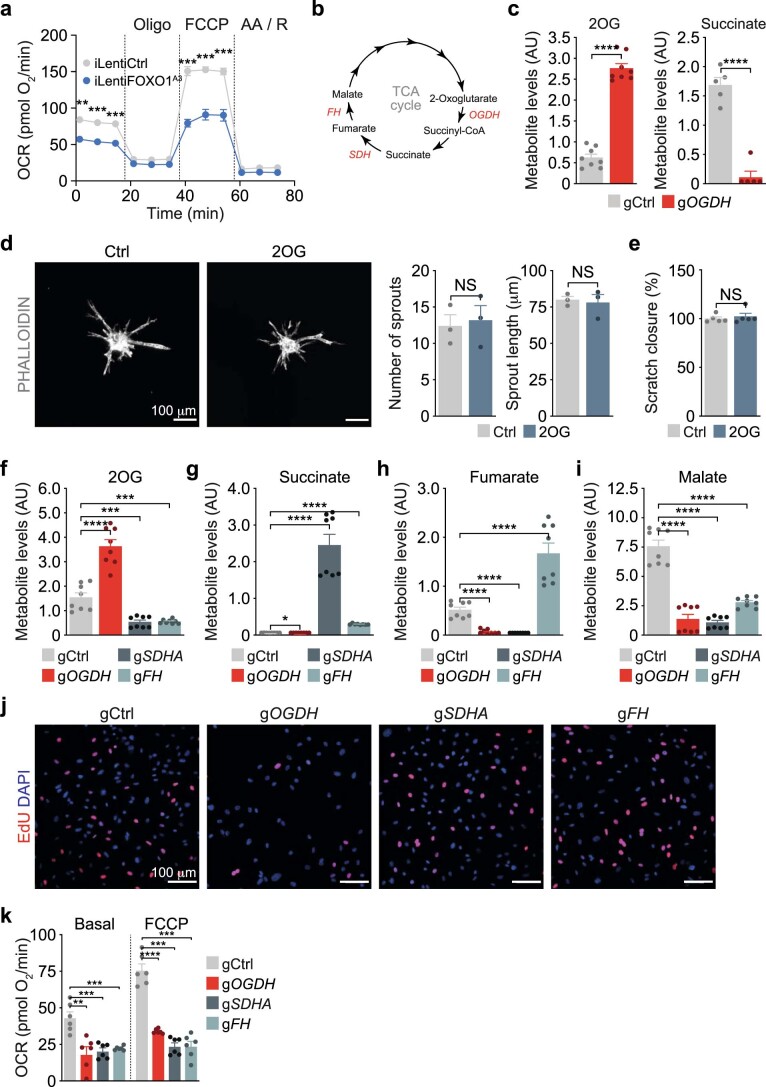
Changes in endothelial mitochondrial metabolism upon FOXO1^A3^ expression or OGDH depletion. **a**, Oxygen consumption rate (OCR) in control (iLentiCtrl) and FOXO1^A3^-transduced (iLentiFOXO1^A3^) HUVECs 48h after doxycycline induction. Oligo, oligomycin; FCCP, fluoro-carbonyl cyanide phenylhydrazone; AA / R, Antimycin A / Rotenone, (n=4 independent samples). **b**, Schematic representation showing the metabolic substrates and products catalysed by the TCA cycle enzymes OGDH, SDH and FH. **c**, Metabolite levels of 2-oxoglutarate (2OG) and succinate in control (g*Ctrl*) and *OGDH*-depleted (g*OGDH*) HUVECs, (2OG: n=8, 8 independent samples for gCtrl and g*OGDH*; Succinate: n=5, 5 for gCtrl and g*OGDH*). AU, arbitrary units. **d**, Confocal images of phalloidin- (grey) labelled HUVEC spheroids showing that 2OG does not affect endothelial sprouting. Images were taken 24h after treatment. Quantifications are shown on the right, (n=3 independent samples). **e**, Scratch-wound assay quantification of vehicle (Ctrl) and 2OG-treated HUVECs, (n=5 independent samples). **f-i**, TCA cycle metabolite levels in control (gCtrl), *SDHA*- (g*SDHA*), *OGDH*- (g*OGDH*) and *FH*-depleted (g*FH*) HUVECs, (n=8 independent samples). AU, arbitrary units. **j**, EdU-incorporation in gCtrl, g*SDHA*, g*OGDH* and g*FH* transduced HUVECs. DAPI was used to identify endothelial nuclei. **k**, Oxygen consumption rate (OCR) in gCtrl, g*OGDH*, g*SDHA* and g*FH* depleted HUVECs. Measurements were performed under basal conditions and in response to FCCP stimulation, (n=6 independent samples). **a**, **c-i** and **k**, Data represent mean ± s.e.m.; a two-tailed unpaired *t*-test was used; ** *P*<0.01; ***P<0.001; **** *P*<0.0001; NS, not significant. The numerical data and *P* values are provided as source data. Source data

Previous studies have shown that *S*-2HG is generated in conditions of reduced mitochondrial function in which 2OG becomes promiscuously reduced to *S*-2HG^33–35,37–41^. To understand how ECs generate *S*-2HG in response to FOXO1 activation, we examined mitochondrial metabolism. This analysis revealed altered mitochondrial respiration in FOXO1^A3^-expressing ECs (Extended Data Fig. 6a), which suggests that there is diminished activity of the 2OG dehydrogenase (OGDH) enzyme—a multisubunit complex that catalyses the conversion of 2OG to succinyl-CoA (Extended Data Fig. 6b). Indeed, we observed that FOXO1^A3^-expressing ECs accumulated the OGDH substrate 2OG, while succinyl-carnitine—a surrogate metabolite of succinyl-CoA—was depleted (Fig. 4a and Supplementary Table 1). Of note, recent genetic studies identified OGDH inhibition as a mechanism for *S*-2HG generation^33,42,43^. We therefore used the clustered regularly interspaced short palindromic repeats (CRISPR)–Cas9 method to specifically inactivate OGDH in ECs (Fig. 4b). Transduction of HUVECs with lentiviral constructs encoding FLAG-tagged Cas9 (Cas9^FLAG^) and guide RNAs (gRNAs) targeting *OGDH* (g*OGDH*) led to the expected accumulation of 2OG and a decline in the mitochondrial oxygen consumption rate (OCR) (Fig. 4c and Extended Data Fig. 6c). Importantly, OGDH deficiency increased endothelial *S*-2HG levels and abrogated the ability of ECs to proliferate (Fig. 4d–g). The growth inhibitory effect was not caused by the increased 2OG levels, because treatment of HUVECs with (cell permeable) 2OG did not influence their capacity to sprout or divide (Fig. 4h and Extended Data Fig. 6d,e). Moreover, it was not a generic response to TCA cycle perturbation, because inactivation of two other TCA cycle enzymes, succinate dehydrogenase (SDH) and fumarate hydratase (FH), neither increased *S*-2HG levels nor inhibited endothelial proliferation (Fig. 4i–k and Extended Data Fig. 6f–k). These data suggest a crucial role of OGDH in endothelial cell-cycle control, which relies on the signalling metabolite *S*-2HG.

### Endothelial OGDH is crucial for angiogenic growth

**Fig. 5:**
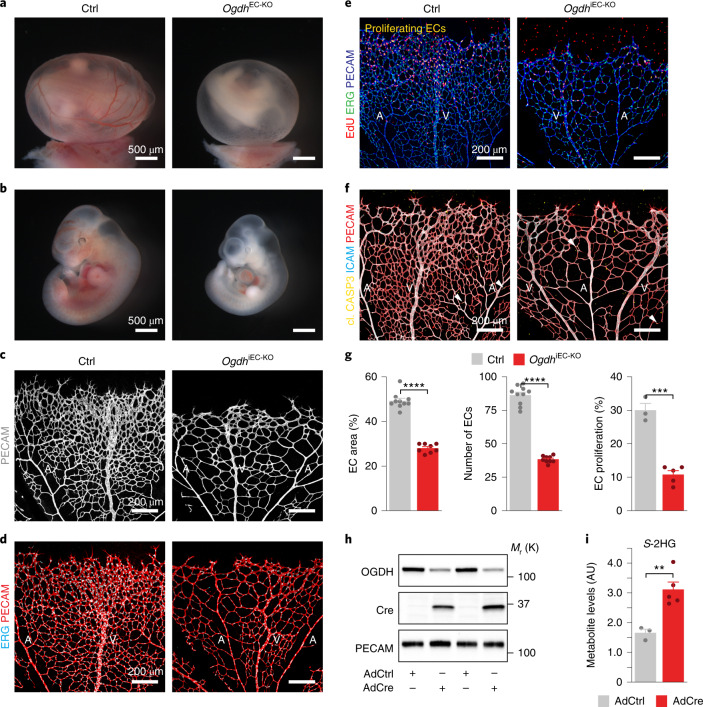
Loss of endothelial OGDH restricts vascular growth. **a**,**b**, Control (*Ogdh*^fl/fl^, Ctrl) and endothelial-restricted *Ogdh* (*Tie2-cre;Ogdh*^fl/fl^, *OGDH*^EC-KO^) mutant yolk sacs (**a**) and embryos (**b**) at embryonic day 11. 5 (E11.5) showing reduced vascularization of the yolk sac and retarded development of the *Ogdh* knockout embryos. **c**, Confocal images of PECAM-stained (grey) P6 retinas isolated from control (*Ogdh*^fl/fl^) and inducible endothelial-specific *Ogdh* mutant mice (*Cdh5-creERT2;Ogdh*^fl/fl^; *Ogdh*^iEC-KO^) following 4-OHT injection from P1 to P4. **d**, Immunofluorescence staining for ERG (cyan) and PECAM (red) in control and *Ogdh*^iEC-KO^ mice. **e**, Labelling of EdU (red), ERG (green) and PECAM (blue) of control and *Ogdh*^iEC-KO^ retinas at P6, revealing a decreased number of proliferating ECs (yellow) in *Ogdh*^iEC-KO^ mutants. **f**, Confocal images of cleaved caspase-3 (yellow), ICAM (cyan) and PECAM (red) labelling in retinas from control and *Ogdh*^iEC-KO^ mice. White arrowheads indicate apoptotic ECs. **g**, Quantification of retinal angiogenesis at P6 in control and *Ogdh*^iEC-KO^ mice (EC area: *n* = 10 (control) and 8 (*Ogdh*^iEC-KO^) samples; number of ECs: *n* = 10 (control) and 9 (*Ogdh*^iEC-KO^) samples; EC proliferation: *n* = 3 (control) and 5 (*Ogdh*^iEC-KO^) samples). **h**, OGDH protein expression in ECs isolated from the lungs of *Ogdh*^fl/fl^ mice followed by transduction with control (AdCtrl) or Cre-encoding (AdCre) adenoviruses. **i**, *S*-2HG metabolite levels in AdCtrl and AdCre-transduced lung ECs derived from *Ogdh*^fl/fl^ mice (*n* = 3 (AdCtrl) and 5 (AdCre) independent samples). Western blot data in **h** are from the respective experiment, processed in parallel, and are representative of at least three independent experiments. For **g** and **i**, the data represent the mean ± s.e.m.; two-tailed unpaired *t*-test, **P < 0.01, ***P < 0.001, ****P < 0.0001. The numerical data, unprocessed western blots and *P* values are provided as source data. Source data

**Extended Data Fig. 7:**
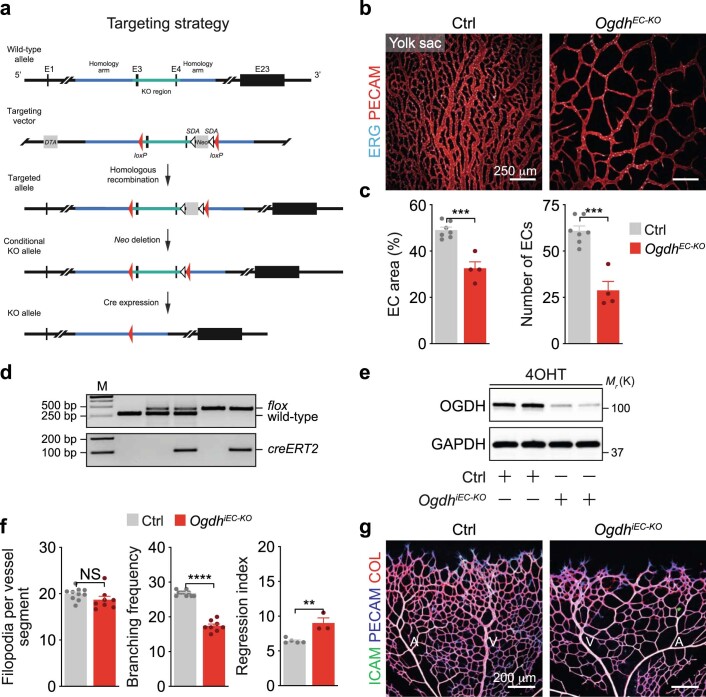
Loss of endothelial *Ogdh* impairs vascular growth. **a**, Schematic illustration of the strategy to generate a conditional *Ogdh* knockout allele, in which exons 3 and 4 are flanked by *loxP* sites (red triangles). The *Ogdh* genomic locus, the targeting vector, the targeted allele and the SDA- and cre-recombined loci are shown. SDA-Neo-SDA, neomycin resistance cassette flanked by SDA sites. **b**, Immunofluorescence staining for ERG (cyan) and PECAM1 (PECAM, red) of control (Ctrl, *Ogdh*^*fl/fl*^) and endothelial-restricted *Ogdh* knockout (*Ogdh*^*EC-KO*^, *Tie2-cre;Ogdh*^*fl/fl*^) yolk sacs at E11.5, showing reduced vascular density after *Ogdh* loss. **c**, Quantifications of vascular parameters in E11.5 Ctrl and *Ogdh*^*EC-KO*^ yolk sacs, as indicated, (EC area: n=7, 4 samples for Ctrl and *Ogdh*^*EC-KO*^; Number of ECs: n=7, 4 samples for Ctrl and *Ogdh*^*EC-KO*^). **d**, PCR analysis of genomic DNA from control (*Ogdh*^*+/+*^, lane 2; *Ogdh*^*fl/+*^, lane 3; *Ogdh*^*fl/fl*^, lane5) and conditional *Ogdh* mutant mice (*Cdh5-creERT2; Ogdh*^*fl/+*^, lane4; *Cdh5-creERT2;Ogdh*^*fl/fl*^, lane 6). Lane 1, DNA marker (M). **e**, Immunoblot analysis of OGDH protein levels in ECs isolated from the liver of 4-OHT-injected Ctrl (*Ogdh*^*fl/fl*^) and *Ogdh*^*iEC-KO*^ (*Cdh5-creERT2;Ogdh*^*fl/fl*^) mouse mutants. GAPDH served as loading control. **f**, Quantifications of vascular parameters in P6 Ctrl and *Ogdh*^*iEC-KO*^ mouse mutants, as indicated (Filopodia per vessel segment: n=10, 8 samples for Ctrl and *Ogdh*^*iEC-KO*^; Branching frequency: n=10, 8 samples for Ctrl and *Ogdh*^*iEC-KO*^; Regression index: n=5, 3 samples for Ctrl and *Ogdh*^*iEC-KO*^). **g**, Immunostaining showing ICAM2- (ICAM, green), PECAM- (blue), and collagen IV- (COL, red) labelling of P6 retinas from 4-OHT-injected Ctrl and *Ogdh*^*iEC-KO*^ mice. Western blot data in **e** were from the respective experiment, processed in parallel, and are representative of at least three independent experiments. **c** and **f**, Data represent mean ± s.e.m.; a two-tailed unpaired *t*-test was used; ** *P*< 0.01; ***P<0.001; ****P<0.0001; NS, not significant. The numerical data, unprocessed western blots and *P* values are provided as source data. Source data

To determine the physiological relevance of these findings, we generated mice in which exon 3 and 4 of the *Ogdh* gene are flanked by *loxP* sites (*Ogdh*^fl^) (Extended Data Fig. 7a). Deleting these exons in ECs (and some haematopoietic cells) using a constitutive *Tie2-cre* transgene (*Ogdh*^EC-KO^) caused severe defects in the yolk sac vasculature that led to developmental retardation and embryonic death (Fig. 5a,b and Extended Data Fig. 7b,c). To further characterize the functional consequences of *Ogdh* deletion, we analysed blood vessel growth in the postnatal retina. To this end, we bred *Ogdh*^fl^ mice with mice expressing the tamoxifen-inducible recombinase creERT2 from the endothelial-restricted *Cdh5* promoter (*Ogdh*^iECKO^). 4-Hydroxytamoxifen (4-OHT)-induced depletion of OGDH caused a sparse vascular network that mimicked the defective vasculature in the constitutive *Ogdh* knockout embryos (Fig. 5c–e and Extended Data Fig. 7d–g). *Ogdh* mutant ECs had elevated *S*-2HG levels and exhibited diminished proliferation, but lacked signs of increased cell death (Fig. 5e–i and Extended Data Fig. 7f,g). This result underscores the importance of OGDH function for endothelial proliferation and vascular growth control.

### FOXO1 inhibits OGDH function via the generation of BCAA catabolites

**Fig. 6:**
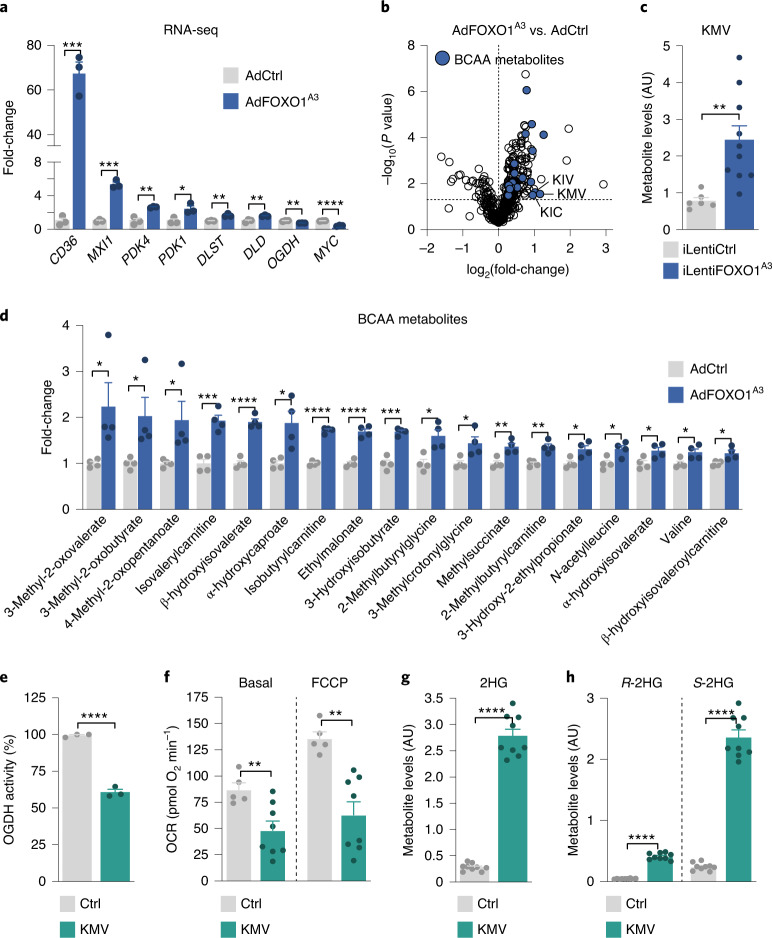
FOXO1 induces *S*-2HG generation by regulating BCAA catabolism. **a**, Expression of canonical FOXO1 targets and OGDH complex subunits in HUVECs transduced with a control-encoding (AdCtrl) and FOXO1^A3^-encoding (AdFOXO1^A3^) adenovirus. Cells were collected 24 h after transduction and analysed by RNA-seq (*n* = 3 independent samples). **b**, Volcano plot showing increased levels of BCAA catabolites in FOXO1^A3^-expressing HUVECs (*n* = 4 independent experiments). **c**, KMV metabolite levels in HUVECs transduced with a doxycycline-inducible control-encoding (iLentiCtrl) or FOXO1^A3^-encoding lentivirus (iLentiFOXO1^A3^) (*n* = 6 (iLentiCtrl) and 10 (iLentiFOXO1^A3^) independent samples). **d**, Changes in BCAA metabolites in AdCtrl versus AdFOXO1^A3^ expressing HUVECs. Data represent the fold-change relative to control (*n* = 4 independent samples). **e**, OGDH activity assay in control (PBS, Ctrl) or KMV-treated HUVECs (*n* = 3 independent experiments). **f**, Decreased basal and maximal (FCCP) OCRs in HUVECs treated with KMV for 48 h compared to control (*n* = 5 (control) or 8 (KMV) independent samples). **g**, 2HG metabolite levels in control and KMV-treated HUVECs measured by LC–MS (*n* = 9 independent samples). **h**, 2HG chiral derivatization and enantioselective MS measurement of *R*- and *S*-2HG levels in control or KMV-treated HUVECs (*n* = 9 independent samples). For **a**–**h**, the data represent the mean ± s.e.m.; two-tailed unpaired *t*-test, *P < 0.05, **P < 0.01, ***P < 0.001, ****P < 0.0001. The numerical data and *P* values are provided as source data. Source data

**Extended Data Fig. 8:**
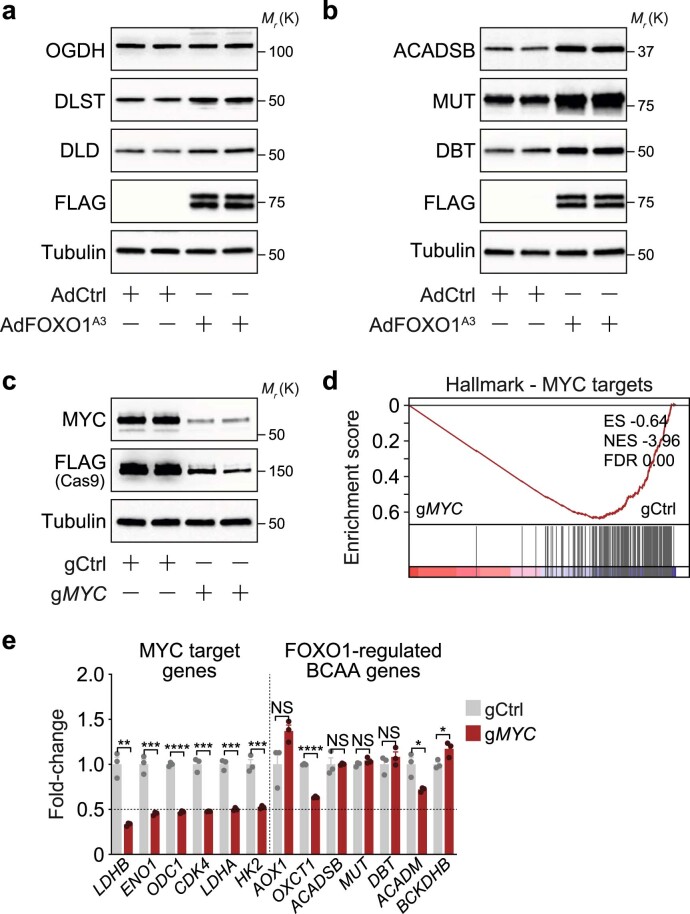
FOXO1 regulates genes involved in BCAA catabolism independent of its suppressive effect on MYC signalling. **a**, Immunoblot analysis in AdCtrl and AdFOXO1^A3^-transduced HUVECs showing the protein expression of the OGDH complex subunits. A FLAG antibody was used to validate the expression of the FLAG-tagged FOXO1^A3^ mutant. Tubulin served as loading control. **b**, Immunoblot analysis of the BCAA metabolism enzymes ACADSB, MUT and DBT in AdCtrl and AdFOXO1^A3^ transduced HUVECs. **c**, Immunoblot analysis of MYC protein levels in control and *MYC*-depleted HUVECs (g*MYC*). Cells were generated by lentiviral transduction with FLAG-tagged Cas9, control (gCtrl) or MYC (g*MYC*) targeting gRNAs. **d**, Gene set enrichment analysis (GSEA) showing a suppression of MYC-regulated genes in g*MYC* HUVECs when compared to controls (*gCtrl*). ES, enrichment score; NES, normalized enrichment score; FDR, false discovery rate. **e**, Expression of canonical MYC target genes and FOXO1-regulated BCAA genes in *gCtrl* and g*MYC* HUVECs. Analysis was performed by RNA-seq, (n=3 independent samples). Western blot data in **a**-**c** were from the respective experiment, processed in parallel, and are representative of at least three independent experiments. Data in **e** represent mean ± s.e.m.; a two-tailed unpaired *t*-test was used unless otherwise indicated; *P<0.05; **P< 0.01; ***P<0.001; ****P<0.0001; NS, not significant. The numerical data, unprocessed western blots and *P* values are provided as source data. Source data

We then investigated the mechanisms through which FOXO1 regulates OGDH function. First, we examined whether FOXO1 regulates the expression of components of the OGDH complex. For this purpose, we performed RNA-seq analysis in control and FOXO1^A3^-expressing HUVECs. While FOXO1 governed the expression of prototypical FOXO1 target genes in the expected manner, we failed to detect changes in components of the OGDH complex that would explain a reduction in enzymatic activity (Fig. 6a). Similar data were obtained at the protein level (Extended Data Fig. 8a). We therefore considered alternative mechanisms of regulation. Our initial metabolomics analysis showed that, besides 2HG, FOXO1 induces the accumulation of 3-methyl-2-oxovalerate (KMV), 3-methyl-2-oxobutyrate (KIV) and 4-methyl-2-oxopentanoate (KIC), and other intermediates of BCAA catabolism (Fig. 6b–d). Strikingly, these metabolites are known inhibitors of OGDH^34,38,44,45^, which suggests that the FOXO1-induced increase in these branched chain α-keto acids (BCKAs) leads to inhibition of the OGDH complex. Consistent with this model, KMV reduced mitochondrial oxygen consumption and OGDH activity, and led to a 9.7-fold increase in endothelial *S*-2HG levels (Fig. 6e–h).

### Genes involved in BCAA metabolism are direct FOXO1 target genes

**Fig. 7:**
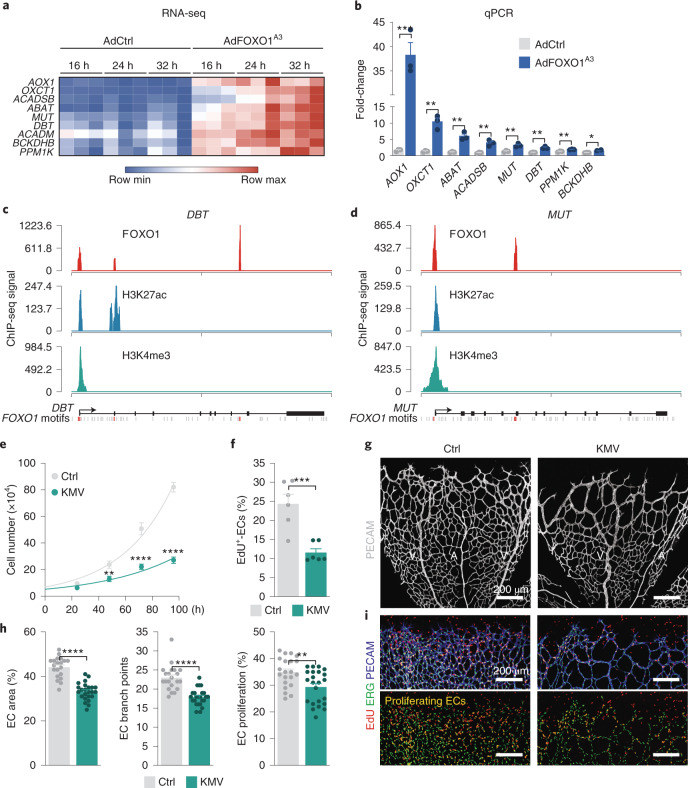
The FOXO1-regulated BCAA catabolite KMV limits endothelial proliferation. **a**, Heatmap of genes involved in BCAA metabolism that are induced in FOXO1^A3^-expressing (AdFOXO1^A3^) HUVECs compared to control (AdCtrl). Transcript levels were assessed at 16, 24 and 32 h post-transduction and analysed by RNA-seq. Genes with a fold-change ≥2 and a *P* value of <0.05 are shown (*n* = 3 independent samples). **b**, RT–qPCR analysis validating the increased expression of BCAA metabolism genes in AdFOXO1^A3^-transduced HUVECs. Values are normalized to β-actin levels and represented as fold-change relative to control (*n* = 3 independent samples). **c**,**d**, FOXO1, H3K27ac and H3K4me3 ChIP-seq signals at the genomic loci of the *DBT* (**c**) and *MUT* (**d**) gene. FOXO consensus motifs bound by FOXO1 are indicated in orange. Unbound FOXO motifs are shown in grey. ChIP-seq signals are represented as reads per kilobase million. **e**, Cell-proliferation curves of HUVECs treated with vehicle (PBS, Ctrl) or KMV for the indicated times (*n* = 12 independent samples). **f**, EdU incorporation is reduced in HUVECs treated with KMV for 48 h (*n* = 6 independent samples). **g**, Confocal images for PECAM (grey) labelling in P7 mouse retinas showing decreased vascular density after a single intraocular injection of KMV at P5. Mice injected with PBS were used as a control. **h**, Quantifications of vascular parameters in the retina of control and KMV-injected mice, as indicated (EC area: *n* = 20 (control) and 24 (KMV) samples; EC branch points: *n* = 21 (control) and 24 (KMV) samples; EC proliferation: *n* = 21 (control) and 23 (KMV) samples). **i**, EdU (red), ERG (green) and PECAM (blue) labelling of control and KMV-injected retinas at P7, revealing decreased endothelial proliferation following KMV treatment. For **b**, **e**, **f** and **h**, the data represent the mean ± s.e.m.; two-tailed unpaired *t*-test, *P < 0.05, **P < 0.01, ***P < 0.001, ****P < 0.0001. The numerical data and *P* values are provided as source data. Source data

We probed further to elucidate how FOXO1 regulates BCKA generation. Interrogation of the transcriptional signature of FOXO1^A3^-transduced HUVECs demonstrated that FOXO1 induced several genes coding for enzymes of the BCAA catabolic pathway, including dihydrolipoamide branched chain transacylase E2 (*DBT*), branched chain keto acid dehydrogenase E1 subunit beta (*BCKDHB*), acyl-CoA dehydrogenase short/branched chain (*ACADSB*) and methylmalonyl-CoA mutase (*MUT*), among others (Fig. 7a,b and Extended Data Fig. 8b). Notably, *DBT* and *BCKDHB* are subunits of the branched-chain α-ketoacid dehydrogenase (BCKD) complex, which oxidizes KMV, KIC and KIV to branched-chain acyl-CoAs and constitutes the rate-limiting step in BCAA metabolism^46^. Moreover, expression of protein phosphatase, Mg^2+^/Mn^2+^-dependent 1K (*PPM1K*), a phosphatase that activates the BCKD complex^46^, was also increased by FOXO1, which suggests that FOXO1 coordinates the expression of genes involved in BCAA catabolism. Of note, FOXO1 regulates these genes independently of its repressive effects on MYC signalling^4^, since inactivation of MYC by CRISPR–Cas9 did not suppress the levels of these BCAA transcripts (Extended Data Fig. 8c–e).

**Extended Data Fig. 9:**
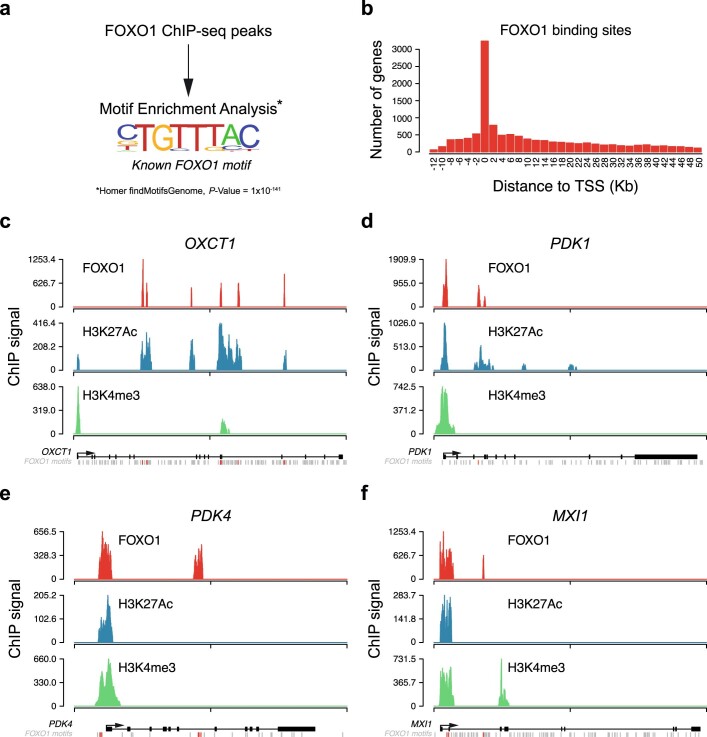
Identification of FOXO1 target genes by ChIP-seq. **a**, Genome-wide analysis of FOXO1 binding peaks revealed the FOXO1 high-affinity binding sequence as the most enriched motif in the immunoprecipitated chromatin of FOXO1^A3^-transduced HUVECs. **b**, Distribution of FOXO1 bindings peaks relative to the transcriptional start site (TSS), showing preferential binding of FOXO1 to genomic regions located at gene promoters near the TSS. **c-f**, ChIP-seq signals for FOXO1, H3K27Ac and H3K4me3 at the genomic loci of (**c**) *OXCT1*, (**d**) *PDK1*, (**e**) *PDK4* and (**f**) *MXI1*. The FOXO consensus motifs that are bound by FOXO1 are highlighted in orange. ChIP-seq signals are represented as reads per kilobase million (RPKMs).

We therefore studied whether FOXO1 directly targets these genes, and conducted FOXO1 chromatin immunoprecipitation with sequencing (ChIP-seq) in FOXO1^A3^-expressing ECs. We found that FOXO1 peaks were enriched for the high-affinity FOXO1-binding motif and were preferentially located at gene promoters close to the transcriptional start site (Extended Data Fig. 9a,b). FOXO1 ChIP-seq identified bona fide FOXO1 targets, including several of the differentially regulated BCAA genes (*DBT*, *DLD*, *PPM1K*, *MUT* and *OXCT1*) (Fig. 7c,d, Extended Data Fig. 9c–f and Supplementary Table 3). These FOXO1-bound genes were also in an active state, as indicated by the acetylation of H3K27 and the trimethylation of H3K4 (Fig. 7c,d and Extended Data Fig. 9c–f). These results demonstrate that FOXO1 drives a transcriptional programme involved in BCAA catabolism and suggest a mechanism for the generation of BCKAs such as KMV.

### BCAA metabolites restrict endothelial proliferation and vascular growth

Finally, we assessed the impact of BCKAs on ECs by treating HUVECs with KMV. Similar to *S*-2HG, KMV suppressed the proliferative activity of ECs without affecting their viability (Fig. 7e,f). Intraocular injection of KMV in neonatal pups (at P5) also reduced endothelial proliferation, giving rise to a sparse vascular network that resembled the vasculature in *S*-2HG-treated mice (Fig. 7g–i). Although we cannot exclude that KMV might also signal via alternative pathways, these data strongly suggest a pivotal role for KMV (and other BCKAs) in FOXO1-induced quiescence signalling.

## Discussion

In summary, we identified a network of FOXO1-regulated metabolites that promote the acquisition of a quiescent endothelial state, and in which the metabolic signalling molecule *S*-2HG plays a critical role. We propose that endothelial *S*-2HG levels increase in response to FOXO1 activation to induce a potent but reversible cell cycle arrest. Our data suggest that FOXO1 stimulates *S*-2HG generation by increasing BCKAs such as KMV, which inhibit the activity of the mitochondrial OGDH complex. Inhibition of this TCA cycle enzyme results in the accumulation of its substrate 2OG, which is subsequently reduced to *S*-2HG via promiscuous substrate usage^34,35,37,38^. Changes in endothelial FOXO1 activity therefore determine whether 2OG is used in the TCA cycle to support EC proliferation or is converted to *S*-2HG to signal quiescence. Our data are consistent with studies of several cancer cell lines showing that OGDH activity is necessary for cell proliferation^47^ and that *S*-2HG can mount antiproliferative responses^48,49^.

**Extended Data Fig. 10:**
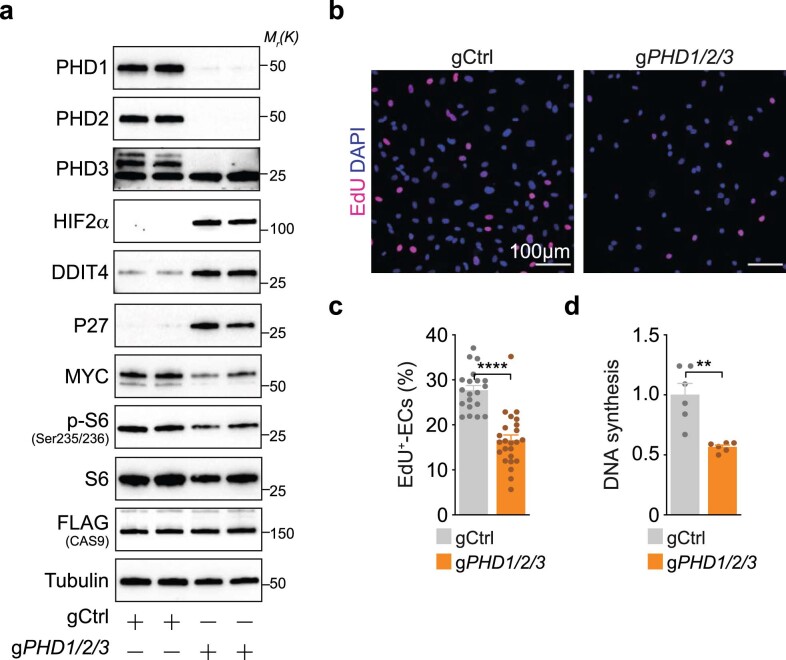
Genetic PHD inactivation lowers the proliferative activity of ECs. **a**, Immunoblot analysis of PHD1/2/3-depleted HUVECs showing regulation of HIF targets and proliferation-associated proteins. Cells were generated by lentiviral transduction with FLAG-tagged Cas9, control- (*gCtrl*) or *PHD1/2/3*-targeting gRNAs (g*PHD1/2/3*). **b**, EdU-incorporation in PHD1/2/3-depleted HUVECs. DAPI was used to identify ECs nuclei. **c**, Quantification of EdU-incorporation in gCtrl and g*PHD1/2/*3-transduced HUVECs. Values represent the percentage of EdU-labelled ECs, (n=20, 24 independent samples for gCtrl and g*PHD1/2/3*). **d**, Reduced DNA synthesis in PHD1/2/3-depleted ECs, as assessed by analysing ^3^H-thymidine incorporation. Values are represented as fold-change relative to control, (n=6 independent samples). Western blot data in **a** were from the respective experiment, processed in parallel, and are representative of at least three independent experiments. **c** and **d** represent mean ± s.e.m.; a two-tailed unpaired *t*-test was used; **P< 0.01; ****P<0.0001. The numerical data, unprocessed western blots and *P* values are provided as source data. Source data

The precise mechanisms by which *S*-2HG limits endothelial cell-cycle progression remain to be addressed. We found that *S*-2HG leads to increased HIF abundance and a robust HIF transcriptional response, which is in accordance with the inhibitory function of *S*-2HG on PHD enzymes^30,31,34,35^ and the cell-cycle-arresting activity of HIFs^50,51^. Importantly, partial inactivation of PHD2 in ECs has also been shown to cause a resting (phalanx) phenotype via stabilization of HIF proteins^52^, which suggests that PHDs are quantitatively sensitive targets of *S*-2HG that may contribute to its endothelial effects. In line with these reports, we found that genetic PHD inactivation lowers the proliferative capacity of ECs (Extended Data Fig. 10).

Yet, *S*-2HG inhibits a variety of other 2OG-dependent dioxygenases, including JmjC histone lysine demethylases, ten-eleven translocation (TET) DNA hydroxylases and members of the AlkB family of proteins, which control histone, DNA and RNA methylation, respectively^33,37–39,48,53^. Although the vascular functions of these epigenetic regulators are largely unknown, it seems probable that they contribute to the effects of *S*-2HG in ECs. Future studies should aim to elucidate the role of these enzymes in the endothelium and assess their regulation by *S*-2HG and other small-molecule metabolites. Understanding such metabolic signalling mechanisms will provide insight into how blood vessels develop and are maintained, and how their functions become abnormal in disease states.

## Methods

### Cells and cell culture

Pooled HUVECs were purchased from Lonza (CC-2519) and cultured in endothelial basal medium (EBM; Lonza) supplemented with hydrocortisone (1 µg ml^−1^), bovine brain extract (12 µg ml^−1^), gentamicin (50 µg ml^−1^), amphotericin B (50 ng ml^−1^), human recombinant epidermal growth factor (10 ng ml^−1^) and 10% fetal bovine serum (FBS; Life Technologies). Human embryonic kidney cells (HEK293FT) were purchased from Life Technologies (R70007) and cultured in DMEM supplemented with 10% FBS (Life Technologies) and geneticin (500 µg ml^−1^; Invitrogen). Cells were tested negative for mycoplasma and maintained at 37 °C in a humidified atmosphere with 5% CO_2_.

The isolation of mouse lung ECs was performed as previously described^4,54^. In brief, lungs from adult *Ogdh*^fl/fl^ mice were washed with ice-cold Hanks buffer (Gibco, 14175-053), dissociated into smaller fragments and incubated with dispase II (Gibco, 17105-041). The homogenate was filtered through a cell strainer, collected by centrifugation and washed with PBS (Gibco, 14190-094) containing 0.1% BSA (PBSB). The resulting cell suspension was incubated with rat anti-mouse VE-cadherin antibody-coated (BD Pharmingen, 555289) magnetic beads (Dynabeads, Invitrogen, 11035). Beads were washed with PBSB and then resuspended in D-MEM/F12 (Invitrogen) supplemented with 20% fetal calf serum (FCS), endothelial growth factor (Promocell, c-30140), penicillin and streptomycin. The isolated cells were seeded on gelatin-coated culture dishes and repurified with the VE-cadherin antibody during the first three passages.

### Cell treatments

Cell-permeable (2*R*)-octyl-α-hydroxyglutarate (*R*-2HG; 16366) and (2*S*)-octyl-α-hydroxyglutarate (*S*-2HG; 16367) from Cayman Biochemical were reconstituted in dimethylsulfoxide (DMSO; Sigma, D4540). Cell-permeable 2OG was obtained from Sigma (349631). For most studies, HUVECs were treated with 800 µM of *R*-2HG, *S*-2HG or 2OG, and DMSO was used as a vehicle control^35,37,39,48^. (±)-3-Methyl-2-oxovaleric acid sodium salt (KMV; Sigma, K7125) was reconstituted in PBS and used at 10 mM at the indicated time points. To block the PHD-dependent degradation of HIFs, cells were treated with 1 mM DMOG (Sigma, D3695) for 6 h. To inhibit autophagy, HUVECs were treated overnight with 50 µM chloroquine (Sigma, C6628). Endothelial apoptosis was induced by treating HUVECs with 10 µg ml^−1^ cycloheximide (Merck-Millipore, 239765) and 10 ng ml^−1^ TNFα (TNF; Invitrogen, PHC3015) for 6 h. For cellular senescence studies, HUVECs were exposed to 100 µM hydrogen peroxide (H_2_O_2_) for 1 h. To measure changes in protein synthesis by surface sensing of translation (SUnSET)^55^, HUVECs were pulsed with 1 µg ml^−1^ puromycin (InvivoGen, ant-pr-1) for 15 min. Protein synthesis was blocked by pretreatment with 100 µM cycloheximide (Merck-Millipore, 239765) for 1 h before the puromycin pulse was applied.

### Adenoviral transductions

Subconfluent HUVECs were transduced with custom-made adenoviruses (Vector Biolabs) to overexpress the FLAG-tagged human FOXO1^A3^ (AdFOXO1^A3^) that is constitutively nuclear^4^. Adenoviruses that contained an empty CMV promoter (AdCtrl; 1300, Vector Biolabs) were used as a control. For transductions, HUVECs were incubated in EBM (Lonza) containing 0.1% (v/v) BSA (Sigma, 1595) for 4 h and transduced with AdCtrl or AdFOXO1^A3^ for an additional 4 h in the presence of 8 µg ml^−1^ polybrene (Santa Cruz). Afterwards, HUVECs were washed five times with Hank’s balanced salt solution (Life Technologies) and cultured in EBM with 10% FBS and supplements. Adenoviral transductions of mouse lung ECs isolated from *Ogdh*^fl/fl^ mice were performed with Cre-encoding adenovirus (AdCre, 1045, Vector Biolabs) or the respective control (AdCtrl).

### Lentiviruses and lentiviral transductions

For doxycycline-inducible lentiviral expression of a constitutively nuclear human FOXO1 (FOXO1^A3^), FLAG-tagged FOXO1^A3^ was cloned into pLVX-TetOne-Puro (Clontech). For CRISPR–Cas9 genome editing, gene-specific gRNA sequences (Supplementary Table 4) were cloned into a plentiCRISPRv2 plasmid co-expressing FLAG-tagged Cas9 nuclease and a puromycin-selection marker (Addgene, 52961). The FUCCI lentiviral reporters mCherry-hCdt1(30/120)-pCSII-EF and mVenus-hGeminin(1/110)-pCSII-EF^56^ were obtained from the Laboratory for Cell Function Dynamics, CBS, RIKEN, Japan.

Lentivirus production was performed by co-transfection of HEK293FT cells with pMD2.G (Addgene, 12259), psPAX2 (Addgene, 12260) and transfer plasmids, accordingly. Transfections were carried out using Lipofectamine 2000 transfection reagent (Life Technologies) as previously described^57^. Viruses were collected 48 and 72 h after transfection, filtered through a 0.45-μm filter and incubated with HUVECs for 16 h in the presence of 8 µg ml^−1^ polybrene (Santa Cruz). After transduction, cells were expanded for 48 h and selected with EBM containing 1 µg ml^−1^ puromycin (InvivoGen, ant-pr-1).

### CRISPR–Cas9 genome editing of HUVECs

Gene-specific gRNA sequences were designed using the Genetic Perturbation Platform from the Broad Institute (https://portals.broadinstitute.org/gpp/public/). For each target gene, three independent gRNAs were used and co-transfected with the packaging vectors for lentivirus production. After transduction, puromycin was used to select a pool of CRISPR–Cas9-mediated mutant HUVECs. Knockout efficiency was confirmed by western blot analysis. Single gRNA sequences are provided in Supplementary Table 4.

### Proliferation assays

HUVECs were seeded onto 6-well plates at a density of 25,000 cells per well and left to attach overnight. Next day (0 h), the indicated treatments were added to the culture medium and total cell numbers were counted every 24 h. Cell culture medium was replaced every 48 h. For ^3^H-thymidine incorporation into DNA, HUVECs treated for 48 h with *S*-2HG or vehicle were pulsed with 1 μCi μl^−1^ ^3^H-thymidine (Perkin Elmer) for 6 h before collection. Samples were processed as previously described^18^, and scintillation was measured with a liquid scintillation analyser (Tri-Carb 2810R, Perkin Elmer). Data were normalized to the total protein concentration per sample.

### RNA and protein synthesis assays

RNA synthesis was measured by ^14^C-glucose incorporation into RNA. HUVECs were pulsed with 0.22 μCi μl^−1^ d-(6-^14^C)-glucose (Perkin Elmer) for 48 h before collection. Samples were processed and analysed as previously described^58^. Data were normalized to the total RNA concentration per sample. To measure protein synthesis by ^3^H-tyrosine incorporation into newly synthesized protein, HUVECs were pulsed with 1 μCi μl^−1^ ^3^H-tyrosine (Perkin Elmer) for 6 h before collection. Samples were processed and analysed as previously described^12^. Data were normalized to the total protein concentration per sample.

### Cell cycle analysis by flow cytometry and live-cell imaging

Cell cycle analysis of HUVECs was performed using a BrdU Flow kit (BD Pharmingen BrdU-APC Flow kit, 557892). HUVECs were pulsed with 10 µM BrdU in culture medium for 45 min before cell collection. Samples were further processed according to the manufacturer’s protocol and analysed by flow cytometry on a LSR Fortessa instrument (BD Biosciences). Analysis of endothelial cell-cycle dynamics was performed in HUVECs expressing the FUCCI reporter^56^ using an IncuCyte Live-Cell imaging system (Essen BioScience).

### Western blotting

Cell lysates, subcellular fractionations and western blot analyses were performed as previously described^59^. The antibodies and amounts used are listed in Supplementary Table 5.

### Spheroid assay

HUVECs resuspended in 80% EBM and 20% Methocel (v/v) were seeded onto a 96-well U-bottomed suspension cell culture plate for 24 h. Spheroids were collected, centrifuged at 200 × *g* for 5 min and resuspended in 80% (v/v) Methocel with 20% (v/v) FBS (Life Technologies). The spheroid–Methocel solution was mixed with equal volumes of collagen matrix and seeded onto a 24-well plate. The plate was incubated for 30 min to allow polymerization and then supplemented with 100 µl of EBM with control vehicle (DMSO), 2OG or *S*-2HG. Endothelial sprouting was allowed for 24 h, after which the spheroid-containing gels were washed with PBS and fixed for 30 min at room temperature in 10% formaldehyde. Bright-field images were acquired, and angiogenic parameters assessed from at least five spheroids per condition. Spheroids were further labelled with Phalloidin-iFluor 488 (Abcam, 176753) overnight at 4 °C, and representative *z*-stack images were acquired by confocal microscopy.

### Scratch-wound assay

Scratch-wound assays were performed using an IncuCyte Cell Migration kit (Essen BioScience, 4493). HUVECs (10,000 per well) were plated onto 96-well ImageLock microplates (Essen BioScience, 4379) and cultured overnight in EBM with 10% FBS and supplements. A uniform scratch-wound was generated using an IncuCyte 96-well WoundMaker (Essen BioScience) following the manufacturer’s instructions. Cells were washed three times with culture medium and cultured with EBM with 10% FBS, supplements and treatments. Images were taken every hour until wound closure. The wound area at 0 and 12 h was measured and the results are expressed as percentage of scratch closure.

### Senescence-associated β-galactosidase staining

Senescence-associated β-galactosidase staining was performed using a kit from Cell Signaling Technology (9860S). For each sample, five representative images were used to quantify the number of cells positive for senescence-associated β-galactosidase. H_2_O_2_-treated HUVECs were used as a positive control. Images were acquired using a LSM800 microscope (Zeiss) with a ×20 objective. Volocity (Perkin Elmer), Fiji/ImageJ, Photoshop (Adobe) and Illustrator (Adobe) software were used for image quantification and processing.

### Immunohistochemistry of cell cultures

HUVECs were seeded on glass-bottom culture dishes (Mattek) coated with fibronectin (50 μg ml^−1^) and cultured at 37 °C and 5% CO_2_. To detect FOXO1 subcellular localization, sparse and dense HUVEC cultures were washed and fixed with 4% paraformaldehyde (PFA) for 20 min at room temperature. Permeabilization was performed in 1% (w/v) BSA, 10% (v/v) donkey serum and 0.5% (v/v) Tween-20 in PBS. Cells were labelled with anti-FOXO1 (Cell Signaling Technology, 2880; 1:100), anti-PECAM-1/CD31 (R&D Biosystems, AF3628; 1:200) and 4,6-diamidino-2-phenylindole (DAPI; 1 µg ml^−1^) diluted in incubation buffer (0.5% BSA, 5% donkey serum and 0.25% Tween-20 in PBS). Following washes in PBS with 0.1% (v/v) Tween-20 (PBST), HUVECs were incubated with Alexa-Fluor-conjugated secondary antibodies (Invitrogen; 1:1,000) diluted in incubation buffer for 2 h at room temperature. Cells were washed in PBST, mounted in VectaShield (Vector Laboratories, H-1000) and confocal images acquired using a SP8 confocal microscope (Leica). Labelling of proliferating cells with EdU was performed using a Click-iT EdU imaging kit (Invitrogen). HUVECs were pulsed with 10 µM EdU in culture medium for 3 h before collection and further processed according to the manufacturer’s instructions.

### Metabolomics analyses

Untargeted biochemical profiling of intracellular metabolites was measured using gas chromatography (GC)–MS and LC–MS/MS platforms from Metabolon. Samples were normalized using the cell number and rescaled to set the median to 1. Missing values were imputed with the minimum value, and analyses performed with hierarchical clustering and PCA. For the analysis of 2HG in HUVECs transduced with the doxycycline-inducible lentiviruses, cells were seeded onto 60-mm dishes and treated with 100 ng ml^−1^ doxycycline (Sigma) for 48 h before collection. For studies using sparse and dense cultures, HUVECs were seeded onto 60-mm dishes and cultured in EBM for 10 days (dense cultures). Culture medium was regularly replaced every 2 days. To generate sparse cultures, dense cultures at day 8 were trypsinized and re-seeded to reinitiate proliferation 36 h before metabolite extraction. After the indicated experimental treatments, HUVECs were washed three times with ice-cold PBS (Life Technologies), and metabolites were collected with extraction buffer (50% (v/v) methanol, 30% (v/v) acetonitrile, 20% (v/v) ultrapure water and 100 ng ml^−1^ HEPES). Thereafter, plates were incubated for 15 min over dry ice and methanol, samples collected by scraping onto Eppendorf tubes and incubated on dry ice for an additional 15 min. Tubes were incubated at 4 °C and shaken at 1,400 r.p.m. for 15 min, and metabolite extracts were cleared by centrifugation at 14,000 r.p.m., 4 °C for 10 min. Finally, the supernatants were transferred into autosampler vials and stored at −80 °C before analysis. LC–MS analysis of sample extracts was performed on a QExactive Orbitrap mass spectrometer coupled to a Dionex UltiMate 3000 Rapid Separation LC system (Thermo). The LC system was fitted with a SeQuant Zic-pHILIC (150 × 2.1 mm, 5 μm) with a guard column (20 × 2.1 mm, 5 μm) from Merck. The flow rate was set at 200 µL min^−1^ with the following gradient: 0 min 80% B, 2 min 80% B, 17 min 20% B, 17.1 min 80% B, and a hold at 80% B for 5 min. The mass spectrometer was operated in full MS and polarity-switching mode. Five independent cell cultures were measured for each condition, and samples were randomized to avoid bias in sample analyses due to machine drift. The acquired spectra were analysed using XCalibur Qual Browser and XCalibur Quan Browser software (Thermo Scientific) by referencing to an internal library of compounds. The R packages muma and gplots were used for data visualization. Quantification of 2HG was performed via interpolation of the corresponding standard curve obtained from serial dilutions of dl-α-hydroxyglutaric acid disodium salt (Sigma, 94577) running with the same batch of samples. Chiral derivatization of 2HG metabolite extracts with (+)-*O*,*O*′-diacetyl-l-tartaric anhydride (DATAN; Sigma, 358924) was performed as previously described^38,60^, with some modifications. Briefly, for each sample, 300 μl of metabolite extract was evaporated to dryness at ambient temperature in a Savant SpeedVac concentrator (Thermo Scientific). The residue was resuspended in 75 μl of freshly prepared mixture of 80% acetonitrile/20% acetic acid (v/v) plus 50 mg ml^−1^ DATAN (Acros Organics) and incubated at 75 °C for 45 min. Samples were cooled to room temperature and centrifuged before the addition of 75 μl 80% acetonitrile/20% acetic acid. The derivatized sample (5 μl) was injected into a Acquity UHPLC HSS T3 column (100 × 2.1 mm, 1.8-μm particle size). A Dionex UltiMate 3000 Rapid Separation LC was coupled to a QExactive Orbitrap mass spectrometer, and a gradient elution of 1.5 mM ammonium formate in water (mobile phase A, pH 3.6 adjusted with formic acid) and 0.1% formic acid in acetonitrile (mobile phase B) was used to separate the derivatized *R*- and *S*-2HG enantiomers. The target analytes were monitored by in parallel-reaction monitoring in negative electrospray ionization mode. The transition (precursor ion → product ion) of *m/z* 363 → 147 for derivatized *R*- and *S*-2HG was monitored, and an accurate mass of 147.03 was extracted using XCalibur Qual Browser and XCalibur Quan Browser software (Thermo Scientific) and used for relative quantification. Experimental samples were randomized to avoid bias in sample analyses due to machine drift.

### Metabolic assays

The OCR was measured using a Seahorse XFe96 analyser (Seahorse Bioscience). In brief, HUVECs (40,000 cells per well) were seeded onto fibronectin-coated XFe96 microplates. After 4 h, the medium was changed to a non-buffered assay medium and cells were maintained in a non-CO_2_ incubator for 1 h. Using a Mito Stress test kit (Seahorse Bioscience), the OCR was measured sequentially under basal conditions, after injection of oligomycin (3 µM), the mitochondrial uncoupler carbonyl cyanide-4-(trifluoromethoxy)phenyl-hydrazone (FCCP; 1 µM) and after injection of a mix of the respiratory chain inhibitors antimycin A (1.5 µM) and rotenone (3 µM), as previously described^4^. OGDH activity and ATP levels were measured in HUVEC lysates (1 × 10^6^ cells) using a OGDH activity assay kit (Sigma) and an ATP Bioluminescence Assay kit CLS II (Roche), respectively.

### RNA-seq and GSEA

For RNA-seq, RNA was isolated from HUVECs using a miRNeasy micro kit (Qiagen) combined with on-column DNase digestion (DNase-Free DNase Set, Qiagen). RNA and library preparation integrity were verified using a LabChip Gx Touch 24 (Perkin Elmer). For input, 4 μg of total RNA was used for Truseq Stranded mRNA library preparation following the low-sample protocol (Illumina). Sequencing was performed on a NextSeq500 instrument (Illumina) using v2 chemistry, resulting in a minimum of 32 million reads per library with 1 × 75-bp single-end setup. Processing of raw reads was performed as previously described^61^. For GSEA^62^ and Gene Ontology terms analysis, gene set collections from the Molecular Signatures Database (MSigDB) 4.0 (http://www.broadinstitute.org/gsea/msigdb/) were used. Heatmaps were generated using Morpheus (https://software.broadinstitute.org/morpheus/).

### RT–qPCR

RNA isolation, complementary DNA synthesis and quantitative PCR with reverse transcription (RT–qPCR) were performed as previously described^4^. The human TaqMan Gene Expression assays used are listed in Supplementary Table 6.

### ChIP assay

HUVECs were fixed with 1% formaldehyde for 15 min and quenched with 0.125 M glycine. Chromatin was isolated via the addition of lysis buffer, followed by disruption with a Dounce homogenizer. Lysates were sonicated, and the DNA sheared to an average length of 300–500 bp. Genomic DNA (input) was prepared by treating aliquots of chromatin with RNase, proteinase K and heat for reverse-crosslinking, followed by ethanol precipitation. Pellets were resuspended and the resulting DNA was quantified on a NanoDrop spectrophotometer. Extrapolation to the original chromatin volume allowed quantitation of the total chromatin yield. Sheared chromatin (30 μg) was precleared with protein A agarose beads (Invitrogen). Genomic DNA regions of interest were isolated using 4 μg of ChIP-grade antibodies against FOXO1 (Abcam, ab39670), H3K4me3 (Active Motif, 39159) and H3K27ac (Active Motif, 39133). Complexes were washed, eluted from the beads with SDS buffer and subjected to RNase and proteinase K treatment. Crosslinks were reversed by incubation overnight at 65 °C, and ChIP DNAs were purified by phenol–chloroform extraction and ethanol precipitation.

### ChIP-seq

Illumina sequencing libraries were prepared from ChIP and input DNAs by the standard consecutive enzymatic steps of end-polishing, dA-addition and adaptor ligation. After a final PCR amplification step, the resulting DNA libraries were quantified and sequenced on Illumina’s NextSeq 500 (75-nt reads, single end). Reads were aligned to the human genome (hg38) using the Burrows–Wheeler algorithm^63^ (default settings). Duplicate reads were removed, and only uniquely mapped reads (mapping quality ≥ 25) were used for further analysis. Alignments were extended in silico at their 3′-ends to a length of 200 bp, which is the average genomic fragment length in the size-selected library, and assigned to 32-nt bins along the genome. The resulting histograms (genomic ‘signal maps’) were stored in bigWig files. Peak locations were determined using the MACS algorithm (v.2.1.0)^64^ with a cut-off of *P* value of 1 × 10^−7^. Peaks that were on the ENCODE blacklist of known false ChIP-seq peaks were removed. Signal maps and peak locations were used as input data to Active Motifs proprietary analysis program, which creates tables containing detailed information on sample comparison, peak metrics, peak locations and gene annotations. Binding motifs were identified with the findMotifsGenome program of the HOMER package^65^ using default parameters and input sequences comprising ±100 bp from the centre of the top 1,000 peaks. Individual profiles were produced with a window of 5 bp. All profiles were plotted on a normalized reads-per-million basis. The processed data were plotted and visualized using software of the R project for statistical computing.

### Animals, genetic experiments and intraocular injections

The conditional *Ogdh* knockout allele was generated by flanking exons 3 and 4 with *loxP* sites. Loss of exons 3 and 4 after Cre-mediated recombination results in a frameshift of the *Ogdh* gene. Mice were on a C57BL/6 genetic background and were generated by Cyagen Biosciences. For constitutive Cre-mediated recombination in ECs, *Ogdh*^fl/fl^ mice (*Ogdh*^EC-KO^) were bred with *Tie2-cre* transgenic mice^66^. To avoid recombination in the female germline, only *Tie2-cre*-positive male mice were used for intercrosses. Embryos were collected from *cre*-negative females at the indicated time points and genotyping performed from isolated embryos. For inducible Cre-mediated recombination in ECs, *loxP*-flanked *Ogdh* mice (*Ogdh*^fl/fl^) were bred with transgenic mice expressing the tamoxifen-inducible, *Cdh5* promoter-driven *creERT2* (ref. ^67^) (*Ogdh*^iEC-KO^). For the analysis of angiogenesis in the postnatal mouse retina, Cre-mediated recombination was induced by intraperitoneal injections of 25 µl 4-OHT (2 mg ml^−1^; Sigma) from P1 to P4. Eyes were collected at P6 for analysis. Control animals were littermate animals without Cre. Mice were genotyped by PCR performed on genomic DNA. Genomic DNA was extracted from tail biopsies using DirectPCR Lysis reagent (Peqlab). Intraocular administration of *S*-2HG (800 µM) and KMV (10 mM) into newborn mouse retinas was performed as previously described^68^. Briefly, a single dose of metabolites (0.25 µl) was injected into the vitreous cavity of P5 mice using a Nanoliter 2000 microinjector (World Precision Instruments). As controls, all pups were injected with *S*-2HG or KMV into the one eyeball and with the vehicle (DMSO or PBS, respectively) into the contralateral eye. Eyes were collected at P7 for further analysis by immunohistochemistry. Male and female mice (C57BL/6) were used for this experiment. All mice were maintained under specific pathogen-free conditions, and animal experiments were conducted in accordance with institutional guidelines and laws, following protocols approved by local animal ethics committees and authorities. The genetic experiments were approved by the Regierungspraesidium Darmstadt and the intraocular injections were performed under approval from the Institutional Animal Care and Use Committee of the Korea Advanced Institute of Science and Technology.

### Immunohistochemistry of murine retinal and yolk sac vasculature

To analyse blood vessel growth in the postnatal retina, whole mouse eyes were fixed in 4% (w/v) PFA on ice for 2.5 h. Eyes were washed in PBS and retinas were dissected and partially cut into four leaflets. After blocking and permeabilization in PBS containing 2% (v/v) FCS, 1% (w/v) BSA and 0.5% (v/v) Tween-20 for 1 h at room temperature, retinas were incubated overnight at 4 °C with the primary antibody diluted in PBS containing 1% (v/v) FCS, 0.5% (w/v) BSA, 0.25% (v/v) Tween-20 and 0.25% (v/v) Triton X-100. Primary antibodies against the following proteins were used: collagen IV (Bio-Rad, 2150-1470; 1:400); cleaved (Asp175)-caspase3 (Cell Signaling Technology, 9664; 1:100); ERG (Abcam, ab92513; 1:200); ICAM2 (BD Biosciences, 553326; 1:200); and PECAM-1/CD31 (R&D Biosystems, AF3628; 1:200). After four washes in PBST, retinas were incubated with Alexa-Fluor 488-, Alexa-Fluor 555-, Alexa-Fluor 594- or Alexa-Fluor 647-conjugated secondary antibodies (Life Technologies; 1:400) diluted in blocking buffer for 2 h at room temperature. After four washes in PBST, retinas were flat-mounted with ProLong Gold Antifade reagent (Life Technologies) and imaged by high-resolution confocal microscopy (Leica SP8). Labelling of proliferating cells with EdU was performed using a Click-iT EdU imaging kit (Invitrogen). Briefly, mouse pups were intraperitoneally injected with 25 µl of EdU (6 mg ml^−1^; Invitrogen) for 3 h before retinal collection, and EdU labelling was performed according to the manufacturer’s instructions.

For the analysis of the murine yolk sac vasculature, yolk sacs were flat-pinned on a silicon plate and fixed in 4% PFA on ice for 1 h followed by two washes in PBST. After blocking in 2% (v/v) donkey serum, 0.2% (w/v) BSA in PBST for 2 h at room temperature, the yolk sacs were incubated overnight at 4 °C with the indicated primary antibody diluted in blocking buffer. After five washes in PBST, yolk sacs were incubated with Alexa-Fluor-conjugated secondary antibodies for 2 h at room temperature. Samples were washed four times with PBST, flat-mounted in ProLong Gold Antifade and imaged by confocal microscopy.

Immunostainings were carried out in tissues from littermates and processed under the same conditions. Volocity (Perkin Elmer), Fiji/ImageJ, Photoshop (Adobe) and Illustrator (Adobe) software were used for image acquisition and processing. For all of the images in which the levels of immunostaining were compared, settings for laser excitation and confocal scanner detection were kept constant between groups.

### Quantitative analysis of the vasculature

All quantifications were done on high-resolution confocal images of thin *z*-sections of the sample using Volocity (Perkin Elmer) software. Endothelial coverage and the number of endothelial branch points were quantified behind the angiogenic front in a region between an artery and a vein. All parameters were quantified from a minimum of three vascularized fields per sample. Endothelial coverage was determined by assessing the ratio of the PECAM-positive area to the total area of the vascularized field (400 × 200 μm for murine retinas and 200 × 200 μm for yolk sac vasculature) and expressed as the percentage of the area covered by PECAM-positive ECs. The number of ECs (ERG-positive cells per field), the rate of proliferating ECs (EdU/ERG double-positive cells normalized to the number of ERG-positive cells) or vessel regression (number of ICAM2-negative, collagen-IV-positive basement membrane sleeves per 1,000-μm vessel segment) were also quantified in the same fields. The number of filopodial extensions was quantified at the angiogenic front as previously described^69^. All images shown are representative of the vascular phenotype observed in samples from at least three distinct litters per group.

### Statistics and reproducibility

Statistical analysis was performed using unpaired, two-tailed Student’s *t*-test unless mentioned otherwise. For all bar graphs, data are represented as the mean ± s.e.m. *P* values of <0.05 were considered significant. All calculations were performed using Prism 8.0 (GraphPad Software).

No randomization or blinding was used for the analyses. Images are representative of at least three independent experiments in mice or cells of the same treatment group or genotype. Western blot data were from the respective experiment, processed in parallel and are representative of at least three independent experiments. Sample sizes were selected on the basis of published protocols^69^ and previous experiments.

### Reporting Summary

Further information on research design is available in the Nature Research Reporting Summary linked to this article.

## Online content

Any methods, additional references, Nature Research reporting summaries, source data, extended data, supplementary information, acknowledgements, peer review information; details of author contributions and competing interests; and statements of data and code availability are available at 10.1038/s41556-021-00637-6.

## Supplementary information

Reporting Summary

Supplementary Tables Supplementary Table 1: metabolite changes induced by FOXO1 activation in HUVECs. Supplementary Table 2: genome-wide transcriptome analysis comparing control and *S*-2HG-treated HUVECs for 24 h. Supplementary Table 3: genomic localization of FOXO1-binding sites at the genomic loci of FOXO1 target genes. Supplementary Table 4: gene-specific gRNA sequences used for CRISPR–Cas9 genome-editing studies in HUVECs. Supplementary Table 5: antibodies used for western blot studies. Supplementary Table 6: human TaqMan Gene Expression assays used for RT–qPCR analyses.

Supplementary Video 1 Time-lapse image sequences of HUVECs transduced with the FUCCI reporter comparing the cell cycle dynamics in control ECs.

Supplementary Video 2 Time-lapse image sequences of HUVECs transduced with the FUCCI reporter comparing the cell cycle dynamics in *S*-2HG-treated ECs.

Supplementary Video 3 Scratch-wound assay showing the migratory behaviour of control ECs.

Supplementary Video 4 Scratch-wound assay showing the migratory behaviour of *S*-2HG-treated ECs.

## Data Availability

Deep-sequencing (ChIP-seq and RNA–seq) data that support the findings of this study have been deposited in the Gene Expression Omnibus (GEO) under accession code GSE128636. All other data supporting the findings of this study are available from the corresponding author upon reasonable request. Source data are provided with this paper.

## Source data

Source Data Fig. 1 Statistical source data. Source Data Fig. 1 Unprocessed western blots. Source Data Fig. 2 Statistical source data. Source Data Fig. 2 Unprocessed western blots. Source Data Fig. 3 Statistical source data. Source Data Fig. 4 Statistical source data. Source Data Fig. 4 Unprocessed western blots. Source Data Fig. 5 Statistical source data. Source Data Fig. 5 Unprocessed western blots. Source Data Fig. 6 Statistical source data. Source Data Fig. 7 Statistical source data. Source Data Extended Data Fig. 1 Statistical source data. Source Data Extended Data Fig. 1 Unprocessed western blots. Source Data Extended Data Fig. 2 Unprocessed western blots. Source Data Extended Data Fig. 3 Statistical source data. Source Data Extended Data Fig. 3 Unprocessed western blots. Source Data Extended Data Fig. 4 Unprocessed western blots. Source Data Extended Data Fig. 5 Statistical source data Source Data Extended Data Fig. 6 Statistical source data Source Data Extended Data Fig. 7 Statistical source data. Source Data Extended Data Fig. 7 Unprocessed gels/blots. Source Data Extended Data Fig. 8 Statistical source data. Source Data Extended Data Fig. 8 Unprocessed western blots. Source Data Extended Data Fig. 10 Statistical source data. Source Data Extended Data Fig. 10 Unprocessed western blots.

## References

[1] Augustin HG, Koh GY (2017). Organotypic vasculature: from descriptive heterogeneity to functional pathophysiology. Science.

[2] Potente M, Makinen T (2017). Vascular heterogeneity and specialization in development and disease. Nat. Rev. Mol. Cell Biol..

[3] Paik JH (2007). FoxOs are lineage-restricted redundant tumor suppressors and regulate endothelial cell homeostasis. Cell.

[4] Wilhelm K (2016). FOXO1 couples metabolic activity and growth state in the vascular endothelium. Nature.

[5] Salih DA, Brunet A (2008). FoxO transcription factors in the maintenance of cellular homeostasis during aging. Curr. Opin. Cell Biol..

[6] Eijkelenboom A, Burgering BM (2013). FOXOs: signalling integrators for homeostasis maintenance. Nat. Rev. Mol. Cell Biol..

[7] Sengupta A, Chakraborty S, Paik J, Yutzey KE, Evans-Anderson HJ (2012). FoxO1 is required in endothelial but not myocardial cell lineages during cardiovascular development. Dev. Dyn..

[8] Dharaneeswaran H (2014). FOXO1-mediated activation of Akt plays a critical role in vascular homeostasis. Circ. Res..

[9] De Bock K (2013). Role of PFKFB3-driven glycolysis in vessel sprouting. Cell.

[10] Schoors S (2015). Fatty acid carbon is essential for dNTP synthesis in endothelial cells. Nature.

[11] Kim B, Li J, Jang C, Arany Z (2017). Glutamine fuels proliferation but not migration of endothelial cells. EMBO J..

[12] Huang H (2017). Role of glutamine and interlinked asparagine metabolism in vessel formation. EMBO J..

[13] Yu P (2017). FGF-dependent metabolic control of vascular development. Nature.

[14] Kim B (2018). Endothelial pyruvate kinase M2 maintains vascular integrity. J. Clin. Invest..

[15] Stone OA (2018). Loss of pyruvate kinase M2 limits growth and triggers innate immune signaling in endothelial cells. Nat. Commun..

[16] Bruning U (2018). Impairment of angiogenesis by fatty acid synthase inhibition involves mTOR malonylation. Cell Metab..

[17] Vandekeere S (2018). Serine synthesis via PHGDH is essential for heme production in endothelial cells. Cell Metab..

[18] Eelen G (2018). Role of glutamine synthetase in angiogenesis beyond glutamine synthesis. Nature.

[19] Kalucka J (2018). Quiescent endothelial cells upregulate fatty acid β-oxidation for vasculoprotection via redox homeostasis. Cell Metab..

[20] Coller HA, Sang L, Roberts JM (2006). A new description of cellular quiescence. PLoS Biol..

[21] Liu H, Adler AS, Segal E, Chang HY (2007). A transcriptional program mediating entry into cellular quiescence. PLoS Genet..

[22] Schlereth K (2018). The transcriptomic and epigenetic map of vascular quiescence in the continuous lung endothelium. eLife.

[23] van Velthoven CTJ, Rando TA (2019). Stem cell quiescence: dynamism, restraint, and cellular idling. Cell Stem Cell.

[24] Kaelin WG, McKnight SL (2013). Influence of metabolism on epigenetics and disease. Cell.

[25] Metallo CM, Vander Heiden MG (2013). Understanding metabolic regulation and its influence on cell physiology. Mol. Cell.

[26] Ryan DG (2019). Coupling Krebs cycle metabolites to signalling in immunity and cancer. Nat. Metab..

[27] Losman JA, Koivunen P, Kaelin WG (2020). 2-Oxoglutarate-dependent dioxygenases in cancer. Nat. Rev. Cancer.

[28] Xu W (2011). Oncometabolite 2-hydroxyglutarate is a competitive inhibitor of α-ketoglutarate-dependent dioxygenases. Cancer Cell.

[29] Dang L (2009). Cancer-associated *IDH1* mutations produce 2-hydroxyglutarate. Nature.

[30] Chowdhury R (2011). The oncometabolite 2-hydroxyglutarate inhibits histone lysine demethylases. EMBO Rep..

[31] Koivunen P (2012). Transformation by the (*R*)-enantiomer of 2-hydroxyglutarate linked to EGLN activation. Nature.

[32] Losman JA (2013). (*R*)-2-hydroxyglutarate is sufficient to promote leukemogenesis and its effects are reversible. Science.

[33] Burr SP (2016). Mitochondrial protein lipoylation and the 2-oxoglutarate dehydrogenase complex controls HIF1α stability in aerobic conditions. Cell Metab..

[34] Nadtochiy SM (2016). Acidic pH is a metabolic switch for 2-hydroxyglutarate generation and signaling. J. Biol. Chem..

[35] Intlekofer AM (2017). l-2-Hydroxyglutarate production arises from noncanonical enzyme function at acidic pH. Nat. Chem. Biol..

[36] Graham SM, Vass JK, Holyoake TL, Graham GJ (2007). Transcriptional analysis of quiescent and proliferating CD34^+^ human hemopoietic cells from normal and chronic myeloid leukemia sources. Stem Cells.

[37] Intlekofer AM (2015). Hypoxia induces production of l-2-hydroxyglutarate. Cell Metab..

[38] Oldham WM, Clish CB, Yang Y, Loscalzo J (2015). Hypoxia-mediated increases in l-2-hydroxyglutarate coordinate the metabolic response to reductive stress. Cell Metab..

[39] Tyrakis PA (2016). *S*-2-Hydroxyglutarate regulates CD8^+^ T-lymphocyte fate. Nature.

[40] Anso E (2017). The mitochondrial respiratory chain is essential for haematopoietic stem cell function. Nat. Cell Biol..

[41] Weinberg SE (2019). Mitochondrial complex III is essential for suppressive function of regulatory T cells. Nature.

[42] Ni M (2019). Functional assessment of lipoyltransferase-1 deficiency in cells, mice, and humans. Cell Rep..

[43] Bailey PSJ (2020). ABHD11 maintains 2-oxoglutarate metabolism by preserving functional lipoylation of the 2-oxoglutarate dehydrogenase complex. Nat. Commun..

[44] Patel MS (1974). Inhibition by the branched-chain 2-oxo acids of the 2-oxoglutarate dehydrogenase complex in developing rat and human brain. Biochem. J..

[45] Gibson GE, Blass JP (1976). Inhibition of acetylcholine synthesis and of carbohydrate utilization by maple-syrup-urine disease metabolites. J. Neurochem..

[46] Lynch CJ, Adams SH (2014). Branched-chain amino acids in metabolic signalling and insulin resistance. Nat. Rev. Endocrinol..

[47] Ilic N (2017). *PIK3CA* mutant tumors depend on oxoglutarate dehydrogenase. Proc. Natl Acad. Sci. USA.

[48] Su R (2018). *R*-2HG exhibits anti-tumor activity by targeting FTO/m^6^A/MYC/CEBPA signaling. Cell.

[49] Fu X (2015). 2-Hydroxyglutarate inhibits ATP synthase and mTOR signaling. Cell Metab..

[50] Goda N (2003). Hypoxia-inducible factor 1α is essential for cell cycle arrest during hypoxia. Mol. Cell. Biol..

[51] Manalo DJ (2005). Transcriptional regulation of vascular endothelial cell responses to hypoxia by HIF-1. Blood.

[52] Mazzone M (2009). Heterozygous deficiency of PHD2 restores tumor oxygenation and inhibits metastasis via endothelial normalization. Cell.

[53] Chen F (2017). Oncometabolites d- and l-2-hydroxyglutarate Inhibit the AlkB family DNA repair enzymes under physiological conditions. Chem. Res. Toxicol..

[54] Guarani V (2011). Acetylation-dependent regulation of endothelial notch signalling by the SIRT1 deacetylase. Nature.

[55] Schmidt EK, Clavarino G, Ceppi M, Pierre P (2009). SUnSET, a nonradioactive method to monitor protein synthesis. Nat. Methods.

[56] Sakaue-Sawano A (2008). Visualizing spatiotemporal dynamics of multicellular cell-cycle progression. Cell.

[57] Luo W (2021). Arterialization requires the timely suppression of cell growth. Nature.

[58] Cantelmo AR (2016). Inhibition of the glycolytic activator PFKFB3 in endothelium induces tumor vessel normalization, impairs metastasis, and improves chemotherapy. Cancer Cell.

[59] Lim R (2019). Deubiquitinase USP10 regulates Notch signaling in the endothelium. Science.

[60] Struys EA, Jansen EE, Verhoeven NM, Jakobs C (2004). Measurement of urinary d- and l-2-hydroxyglutarate enantiomers by stable-isotope-dilution liquid chromatography-tandem mass spectrometry after derivatization with diacetyl-l-tartaric anhydride. Clin. Chem..

[61] Zhang T (2015). Prmt5 is a regulator of muscle stem cell expansion in adult mice. Nat. Commun..

[62] Subramanian A (2005). Gene set enrichment analysis: a knowledge-based approach for interpreting genome-wide expression profiles. Proc. Natl Acad. Sci. USA.

[63] Li H, Durbin R (2009). Fast and accurate short read alignment with Burrows–Wheeler transform. Bioinformatics.

[64] Zhang Y (2008). Model-based analysis of ChIP-seq (MACS). Genome Biol..

[65] Heinz S (2010). Simple combinations of lineage-determining transcription factors prime *cis*-regulatory elements required for macrophage and B cell identities. Mol. Cell.

[66] Koni PA (2001). Conditional vascular cell adhesion molecule 1 deletion in mice: impaired lymphocyte migration to bone marrow. J. Exp. Med..

[67] Okabe K (2014). Neurons limit angiogenesis by titrating VEGF in retina. Cell.

[68] Lee J (2013). Angiopoietin-1 guides directional angiogenesis through integrin alphavbeta5 signaling for recovery of ischemic retinopathy. Sci. Transl. Med..

[69] Pitulescu ME, Schmidt I, Benedito R, Adams RH (2010). Inducible gene targeting in the neonatal vasculature and analysis of retinal angiogenesis in mice. Nat. Protoc..

