# Supplementary material for: Control of endothelial quiescence by FOXO-regulated metabolites
Source: Nat Cell Biol. 2021 Apr 1;23(4):413–23. doi: 10.1038/s41556-021-00637-6 (PMC8032556; doi:10.1038/s41556-021-00637-6)
Supplement: Supplementary file 1 — Reporting Summary [file 41556_2021_637_MOESM1_ESM.pdf]

nature research

Corresponding author(s): Michael Potente
Last updated by author(s): Jan 13, 2021

Reporting Summary

Nature Research wishes to improve the reproducibility of the work that we publish. This form provides structure for consistency and transparency in reporting. For further information on Nature Research policies, see Authors & Referees and the Editorial Policy Checklist.

Statistics

For all statistical analyses, confirm that the following items are present in the figure legend, table legend, main text, or Methods section.

n/a	Confirmed	
☐	☒	The exact sample size (*n*) for each experimental group/condition, given as a discrete number and unit of measurement
☐	☒	A statement on whether measurements were taken from distinct samples or whether the same sample was measured repeatedly
☐	☒	The statistical test(s) used AND whether they are one- or two-sided<br>*Only common tests should be described solely by name; describe more complex techniques in the Methods section.*
☒	☐	A description of all covariates tested
☒	☐	A description of any assumptions or corrections, such as tests of normality and adjustment for multiple comparisons
☐	☒	A full description of the statistical parameters including central tendency (e.g. means) or other basic estimates (e.g. regression coefficient) AND variation (e.g. standard deviation) or associated estimates of uncertainty (e.g. confidence intervals)
☐	☒	For null hypothesis testing, the test statistic (e.g. *F*, *t*, *r*) with confidence intervals, effect sizes, degrees of freedom and *P* value noted<br>*Give P values as exact values whenever suitable.*
☒	☐	For Bayesian analysis, information on the choice of priors and Markov chain Monte Carlo settings
☒	☐	For hierarchical and complex designs, identification of the appropriate level for tests and full reporting of outcomes
☒	☐	Estimates of effect sizes (e.g. Cohen's *d*, Pearson's *r*), indicating how they were calculated

Our web collection on statistics for biologists contains articles on many of the points above.

Software and code

Policy information about availability of computer code

Data collection	qRT-PCR: StepOnePlus real-time PCR system (Applied Biosystems)<br>RNA- and ChIP-seq: NextSeq500 (Illumina)<br>Seahorse flux analyzer: Seahorse XFe96 analyser (Seahorse Bioscience)<br>Imaging: SP8 confocal microscope (Leica) and IncuCyte System (Essen BioScience)<br>Western-blot: Chemidoc MP Imaging System (Bio-Rad)<br>Scintillation counting: Liquid Scintillation Analyzer Tri-Carb 2810R (Perkin Elmer)<br>Flow cytometry: LSR Fortessa (BD Biosciences)
Data analysis	Graphs: GrahPad Prism (v8.0)<br>RNA- and ChIP-Seq: R project; MACS (v2.1.0); BWA (v0.7.12); bcl2fastq2 (v2.20); Samtools (v0.1.19); BEDtools (v2.25.0); wigToBigWig (v4); HOMER (v4.10.4)<br>Image analysis: Image Lab 5.1 (Biorad), ImageJ/FIJI (v2.0.0-rc-69/1.52p), Adobe Photoshop 2020 (v21.2.3), Adobe Illustrator 2020 (v24.3) and Volocity 6.3 (Perkin Elmer)<br>Flow cytometry: FACSDiva 8.0.1 (BD Biosciences)

For manuscripts utilizing custom algorithms or software that are central to the research but not yet described in published literature, software must be made available to editors/reviewers. We strongly encourage code deposition in a community repository (e.g. GitHub). See the Nature Research guidelines for submitting code & software for further information.

Data

Policy information about availability of data

All manuscripts must include a data availability statement. This statement should provide the following information, where applicable:

- Accession codes, unique identifiers, or web links for publicly available datasets
- A list of figures that have associated raw data
- A description of any restrictions on data availability

The data supporting the findings of this study are available within the paper. Sequencing data have been deposited in NCBI Gene Expression Omnibus under the accession number GSE128636 (https://www.ncbi.nlm.nih.gov/geo/query/acc.cgi?acc=GSE128636).

The following publicly available databases were used:
GSEA pathway analysis: MsigDB (https://www.gsea-msigdb.org/gsea/msigdb);
gRNA design: Genetic Perturbation Platform (https://portals.broadinstitute.org/gpp/public/);

Any additional information required to interpret, replicate or build upon the findings of this study are available from the corresponding author upon reasonable request.

Field-specific reporting

Please select the one below that is the best fit for your research. If you are not sure, read the appropriate sections before making your selection.

☒ Life sciences ☐ Behavioural & social sciences ☐ Ecological, evolutionary & environmental sciences

For a reference copy of the document with all sections, see nature.com/documents/nr-reporting-summary-flat.pdf

Life sciences study design

All studies must disclose on these points even when the disclosure is negative.

Sample size	Sample size for each experiment is indicated in Figure legend. Sample sizes were selected on the basis of published protocols (Pitulesco et al., Nat.Protocols,2010) and previous experiments (Wilhelm et al., Nature,2016; Lim et al., Science, 2019; Luo et al., Nature, 2020). No statistical methods were used to predetermine sample size. Images are representative of at least three independent experiments in mice or cells of the same treatment group or genotype. Western blot data were from the respective experiment, processed in parallel, and are representative of at least three independent experiments.
Data exclusions	No data were excluded in the analyzed samples.
Replication	All experimental findings were reproduced in multiple independent experiments. For each panel, the number of independent experiments or biological replicates is indicated in the figure legend.
Randomization	No statistical methods were used for randomization. Mice and cells were selected for analysis based on their genotype/treatment.
Blinding	Investigators were not blinded since mice and cells were selected for analysis based on their genotype/treatment.

Reporting for specific materials, systems and methods

We require information from authors about some types of materials, experimental systems and methods used in many studies. Here, indicate whether each material, system or method listed is relevant to your study. If you are not sure if a list item applies to your research, read the appropriate section before selecting a response.

Materials & experimental systems

n/a	Involved in the study	
☐	☒	Antibodies
☐	☒	Eukaryotic cell lines
☒	☐	Palaeontology
☐	☒	Animals and other organisms
☒	☐	Human research participants
☒	☐	Clinical data

Methods

n/a	Involved in the study	
☐	☒	ChIP-seq
☐	☒	Flow cytometry
☒	☐	MRI-based neuroimaging

Antibodies

Antibodies used	Primary antibodies for Western blot analysis:

ACADSB (Sigma, HPA041458, rabbit)
cMYC (Cell Signaling Technology, #9402, rabbit)
cleaved (Asp175)-CASPASE 3 (Cell Signaling Technology, #9664, rabbit)
cleaved (Asp214)-PARP (Cell Signaling Technology, #5625, rabbit)
CASPASE 3 (Cell Signaling Technology, #9662, rabbit)
CRE (Merck Millipore, #69050-3, rabbit)
DBT (Sigma, HPA026485, rabbit)
DDIT4 (Proteintech, #10638-1-AP, rabbit)
DLD (Sigma, HPA044849, rabbit)
DLST (Cell Signaling Technology, #5556, rabbit)
FH (Cell Signaling Technology, #4567, rabbit)
Flag M2 (Sigma, #F-3165, 1:4000, mouse)
FOXO1 (Cell SignalingTechnology, #2880, rabbit)
GAPDH (Cell SignalingTechnology, #2118, rabbit)
HIF1α (Cayman Biochemical, #10006421, rabbit)
HIF2α (CellSignaling Technology, #7096, rabbit)
Histone H3 (abcam, #ab1791, rabbit)
Histone H3K9me3(ActiveMotif, #39161, rabbit)
Histone H3K27me3 (Merck Millipore, #07-449, rabbit)
LAMINA/C (Cell Signaling Technology, #2032, rabbit)
LC3A/B (Cell Signaling Technology, #12741, rabbit)
MXI1 (Santa Cruz, #sc-1042, rabbit)
MUT (Proteintech, #17034-1-AP, rabbit)
OGDH (Sigma, HPA020347, rabbit)
PARP (Cell Signaling Technology, #9532, rabbit)
PCNA (BDBiosciences, #610664, mouse)
PECAM (Santa Cruz, #sc-1506, goat)
PHD1 (abcam, #113077, rabbit)
PHD2 (Cell Signaling Technology, #4835, rabbit)
PHD3 (Invitrogen, #PA116526, rabbit)
Phospho(Ser235/236)-S6 Ribossomal protein (Cell SignalingTechnology, #4857, rabbit)
Puromycin (Merck Millipore, MABE343, mouse)
p27/KIP1 (Cell Signaling Technology, #2552, rabbit)
SDHA (Cell Signaling Technology, #5839, rabbit)
S6-Ribossomal protein (Cell Signaling Technology, #2217, rabbit)
Tubulin (Cell Signaling Technology, #2148, rabbit)

Secondary antibodies for Western blot analysis:
anti-rabbit HRP-conjugated (Jackson Immuno Research Labs, 111-035-008, goat)
anti-mouse HRP-conjugated (Jackson Immuno Research Labs, 315-035-003 ,rabbit)
anti-goat HRP-conjugated (Jackson Immuno Research Labs, 305-036-008 ,rabbit)

Primary antibodies for immunohistochemical analysis:
Collagen IV (Bio-Rad, #2150-1470, rabbit)
cleaved(Asp175)-Caspase3 (Cell Signalling Technology, #9664, rabbit)
ERG (Abcam, #ab92513, rabbit)
FOXO1 (Cell SignalingTechnology, #2880, rabbit)
ICAM2 (BD Biosciences, #553326, rat)
PECAM-1/CD31 (R&D Biosystems, #AF3628, goat)

Secondary antibodies for immunohistochemical analysis:
anti-goat IgG, Alexa Fluor 647 (Life technology, #A21447, donkey)
anti-goat IgG, Alexa Fluor 555 (Life technology, #A21432, donkey)
anti-rabbit IgG, Alexa Fluor 488 (Life technology, #A21206, donkey)
anti-rabbit IgG, Alexa Fluor 555 (Life technology, #A31572, donkey)
anti-rabbit IgG, Alexa Fluor 594 (Life technology, #A21207, donkey)
anti-rat IgG, Alexa Fluor 488 (Life technology, #A21208, donkey)

Primary antibodies for MLECs isolation:
anti-mouse VE-Cadherin antibody (BD Pharmingen, #555289, rat)

Staining of endothelial spheroids:
Phalloidin-iFluor 488 (abcam, #176753)

Validation

All antibodies used in this manuscript were obtained from the indicated commercial vendors and have been validated by the respective manufacturer, as described in their website. All antibodies were used in multiple experiments to detect the indicated target protein giving results according to the expected molecular weight, tissue expression pattern and subcellular localization.

Eukaryotic cell lines

Policy information about cell lines

Cell line source(s)	Pooled human umbilical vein endothelial cells (HUVECs) were obtained from Lonza (#CC-2519). Human embryonic kidney cells (HEK293FT) were purchased from LifeTechnologies (#R70007).
Authentication	None of the cell lines were authenticated by us.
Mycoplasma contamination	Cells were tested negative for mycoplasma.
Commonly misidentified lines (See ICLAC register)	No commonly misidentified cell lines were used.

Animals and other organisms

Policy information about studies involving animals; ARRIVE guidelines recommended for reporting animal research

Laboratory animals	All mice used were on a C57BL/6 genetic background and were kept in environmental conditions of 45–65% relative humidity, temperatures of 20-24°C and a 12h-12 h light-dark cycle, with food and water 'ad libitum'. For constitutive Cre-mediated recombination in endothelial cells, Ogdhfl/fl mice (OgdhEC-KO) were bred with Tie2-cre transgenic mice . To avoid recombination in the female germline, only Tie2-cre-positive male mice were used for intercrossing. Embryos were collected from cre-negative females at embryonic day 11.5. For inducible cre-mediated recombination in endothelial cells, Ogdh-floxed mice (OgdhiEC-KO) were bred with transgenic mice expressing the tamoxifen-inducible, Cdh5 promoter-driven creERT2, and analysis was performed on post-natal day 6. Intraocullar injections were performed on C57BL/6 mice at post-natal day 5 and analysis performed at post-natal day 7.
Wild animals	Wild animals were not used in this study.
Field-collected samples	The study did not use field-collected samples.
Ethics oversight	Experiments involving animal experiments were conducted in accordance with institutional guidelines and laws, following protocols approved by local animal ethics committees and authorities. The genetic experiments were approved by the Regierungspraesidium Darmstadt and the intraocular injections were performed under the approval from the Institutional Animal Care and Use Committee of the Korea Advanced Institute of Science and Technology.

Note that full information on the approval of the study protocol must also be provided in the manuscript.

ChIP-seq

Data deposition

☒ Confirm that both raw and final processed data have been deposited in a public database such as GEO.

☒ Confirm that you have deposited or provided access to graph files (e.g. BED files) for the called peaks.

Data access links *May remain private before publication.*	Datasets generated in this study have been deposited in the Gene Expression Omnibus under accession number GSE128636 - https://www.ncbi.nlm.nih.gov/geo/query/acc.cgi?acc=GSE128636.
Files in database submission	Files available in the database submission:<br><br>Processed data files<br>HUVEC_AdControl_FOXO1_ChIPseq.bw<br>HUVEC_AdFOXO1A3_FOXO1_ChIPseq.bw<br>HUVEC_AdControl_H3K27ac_ChIPseq.bw<br>HUVEC_AdFOXO1A3_H3K27ac_ChIPseq.bw<br>HUVEC_AdControl_H3K4me3_ChIPseq.bw<br>HUVEC_AdFOXO1A3_H3K4me3_ChIPseq.bw<br>HUVEC_pooled_input.bw<br><br>Raw files<br>HUVEC_AdControl_FOXO1_ChIPseq.fastq.gz<br>HUVEC_AdFOXO1A3_FOXO1_ChIPseq.fastq.gz<br>HUVEC_AdControl_H3K27ac_ChIPseq.fastq.gz<br>HUVEC_AdFOXO1A3_H3K27ac_ChIPseq.fastq.gz<br>HUVEC_AdControl_H3K4me3_ChIPseq.fastq.gz<br>HUVEC_AdFOXO1A3_H3K4me3_ChIPseq.fastq.gz<br>HUVEC_pooled_input.fastq.gz
Genome browser session (e.g. UCSC)	

http://genome-euro.ucsc.edu/cgi-bin/hgTracks?db=hg38&lastVirtModeType=default&lastVirtModeExtraState=&virtModeType=default&virtMode=0&nonVirtPosition=&position=chr5%3A41695277%2D41906214&hgsid=233368376_79NiNLc9lDF8QnL6WlRDXDmtkA4a

Methodology

Replicates	1; Pooled chromatin from independent transductions.
Sequencing depth	Illumina sequencing libraries were prepared from the ChIP and Input DNAs by the standard consecutive enzymatic steps of end-polishing, dA-addition, and adaptor ligation.<br>After a final PCR amplification step, the resulting DNA libraries were quantified and sequenced on Illumina's NextSeq 500 (75 nt reads, single end).<br><br>HUVEC-AdControl_FOXO1 45053443 reads<br>HUVEC-AdFOXO1A3_FOXO1 47702593 reads<br>HUVEC-AdControl_H3K27Ac 38862996 reads<br>HUVEC-AdFOXO1A3_H3K27Ac 37462857 reads<br>HUVEC-AdControl_H3K4me3 38628403 reads<br>HUVEC-AdFOXO1A3_H3K4me3 39135063 reads<br>HUVEC-Pooled_Input 50079555 reads
Antibodies	ChIP-grade antibodies:<br>FOXO1 (abcam, #ab39670),<br>H3K4me3 (Active Motif, #39159)<br>H3K27ac (Active Motif, #39133)
Peak calling parameters	Peak locations were determined using the MACS algorithm (v2.1.0) with a cutoff of p-value = 1e-7. Peaks that were on the ENCODE blacklist of known false ChIP-Seq peaks were removed.
Data quality	Data quality was assessed with the FastQC quality-control tool for high throughput sequence data.
Software	MACS (v2.1.0)<br>BWA (v0.7.12)<br>bcl2fastq2 (v2.20)<br>Samtools (v0.1.19)<br>BEDtools (v2.25.0)<br>wigToBigWig (v4)

Flow Cytometry

Plots

Confirm that:

☒ The axis labels state the marker and fluorochrome used (e.g. CD4-FITC).

☒ The axis scales are clearly visible. Include numbers along axes only for bottom left plot of group (a 'group' is an analysis of identical markers).

☐ All plots are contour plots with outliers or pseudocolor plots.

☒ A numerical value for number of cells or percentage (with statistics) is provided.

Methodology

Sample preparation	HUVECs cell cycle analysis by flow cytometry was performed with the BrdU Flow Kit (BD Pharmingen BrdU-APC Flow Kit, #557892).
Instrument	LSRFortessa (BD Pharmingen)
Software	BD FACSDiva 8.0.1 (BD Pharmingen)
Cell population abundance	No FACS sorting was performed during this work.
Gating strategy	FSC/SSC were adjusted such that cell population was on scale. Doublets and cell debris were excluded by initial gating based on 7-AAD only controls. The gating strategy is indicated in Extended data figure 3B.

☒ Tick this box to confirm that a figure exemplifying the gating strategy is provided in the Supplementary Information.
