# Supplementary figures and images for: Control of endothelial quiescence by FOXO-regulated metabolites
Source: Nat Cell Biol. 2021 Apr 1;23(4):413–23. doi: 10.1038/s41556-021-00637-6 (PMC8032556; doi:10.1038/s41556-021-00637-6)

Figure 1A

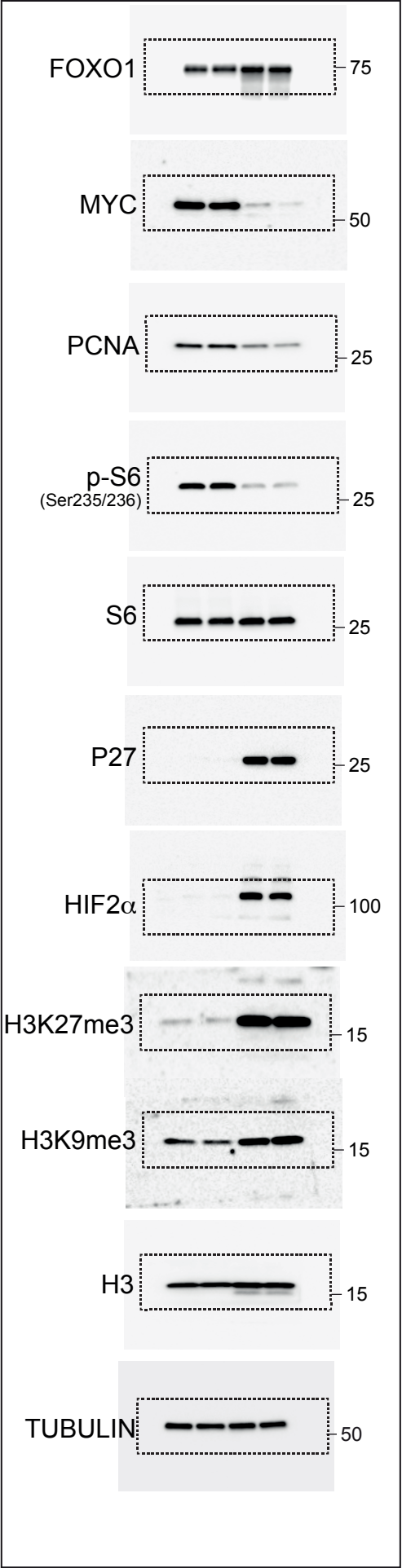

Figure 1H

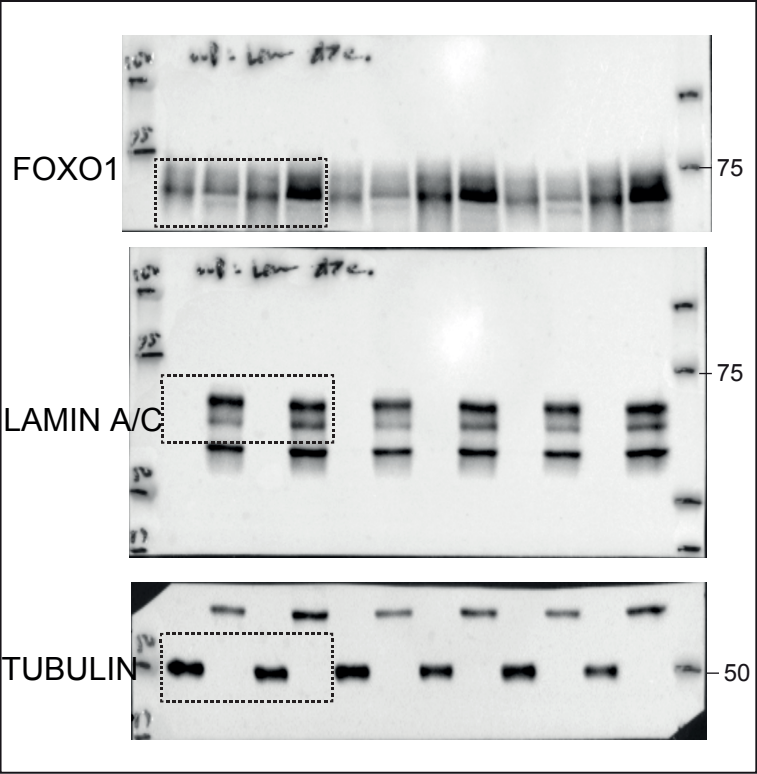

Supplement: Source Data Fig. 1 — Unprocessed western blots. [file 41556_2021_637_MOESM8_ESM.pdf]

Figure 2E

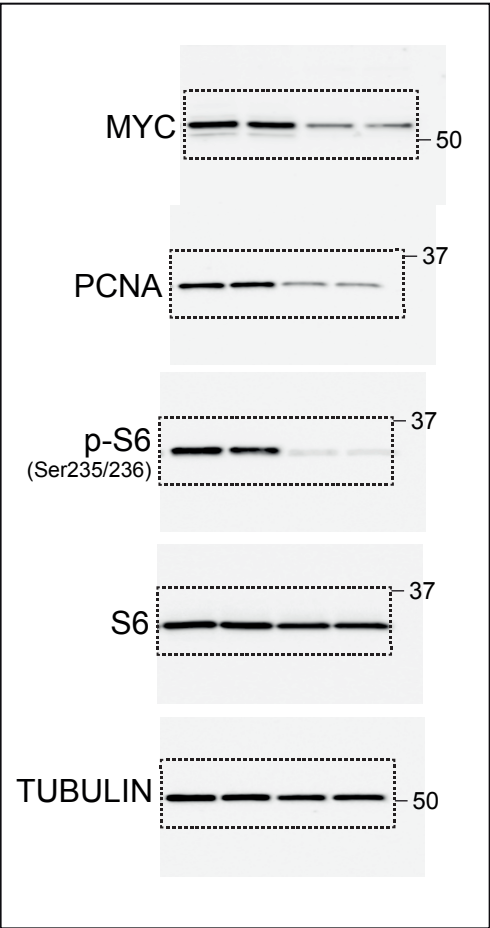

Figure 2F

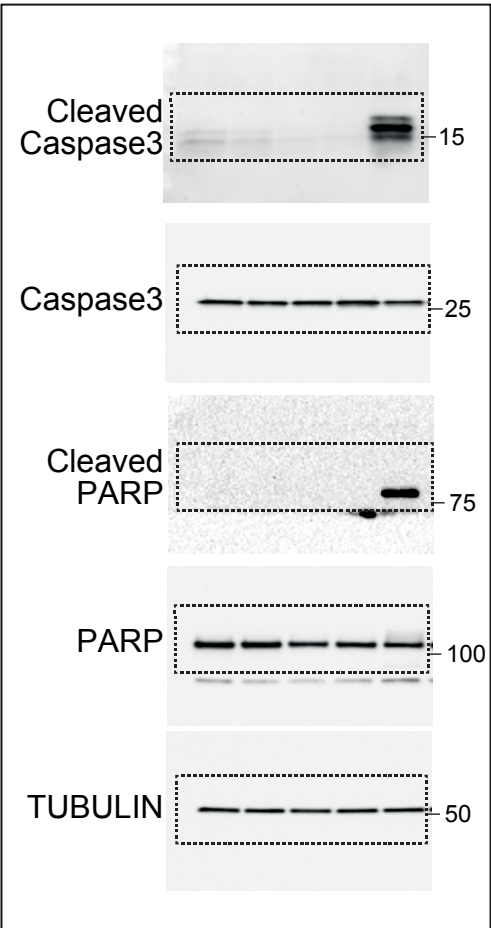

Supplement: Source Data Fig. 2 — Unprocessed western blots. [file 41556_2021_637_MOESM10_ESM.pdf]

Source Data Fig. 4

Figure 4B

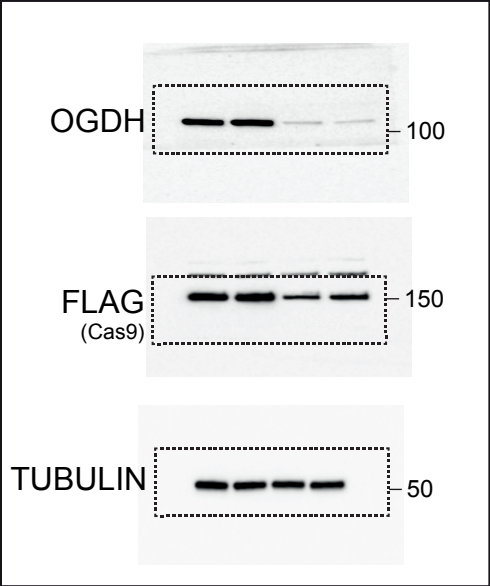

Figure 4I

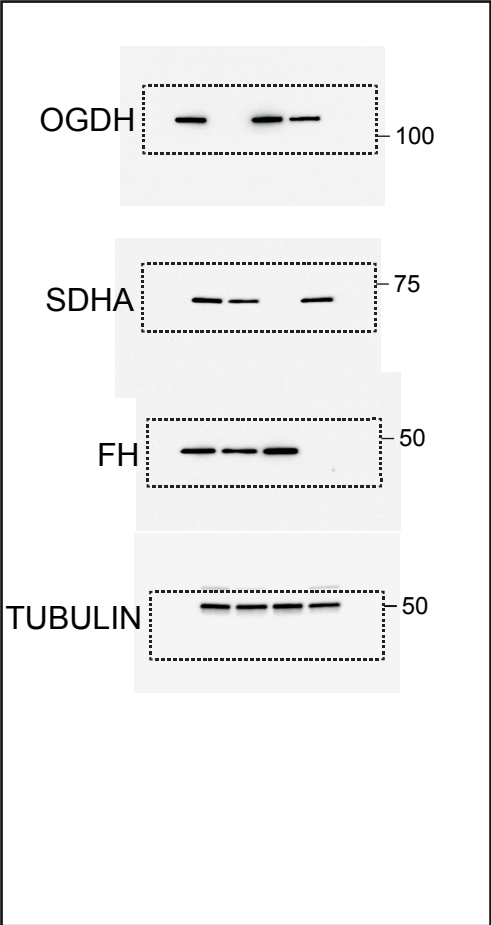

Supplement: Source Data Fig. 4 — Unprocessed western blots. [file 41556_2021_637_MOESM13_ESM.pdf]

Figure 5H

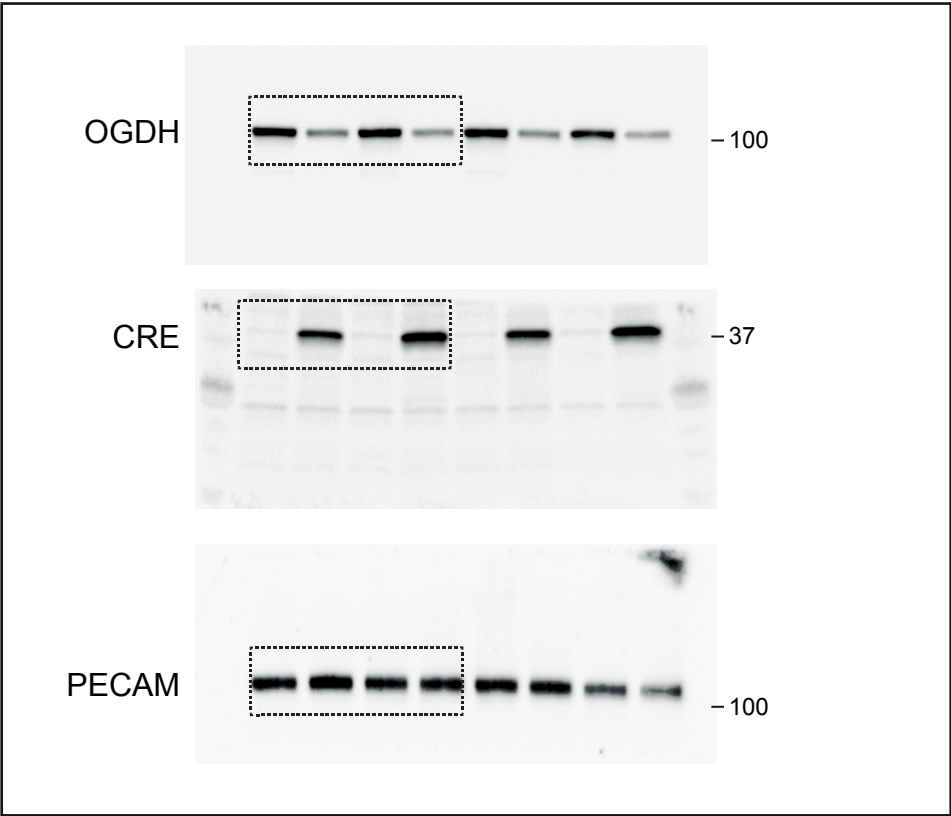

Supplement: Source Data Fig. 5 — Unprocessed western blots. [file 41556_2021_637_MOESM15_ESM.pdf]

Source Data Extended Data Figure 1

Figure 1A

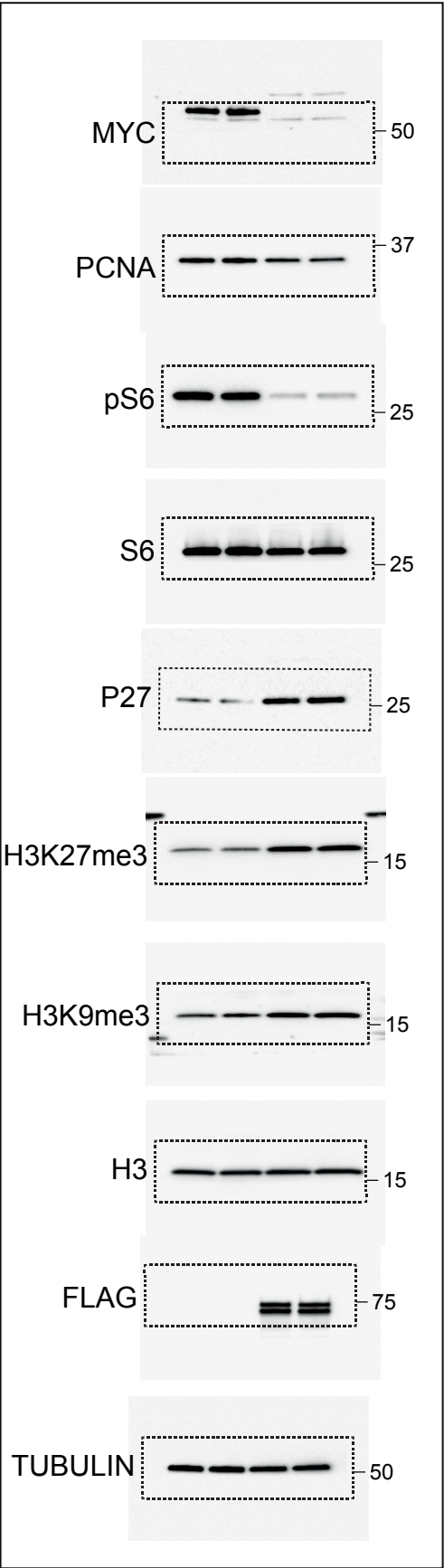

Figure 1E

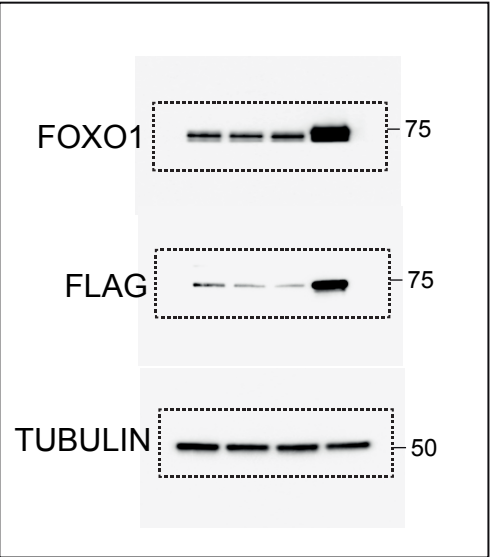

Figure 1F

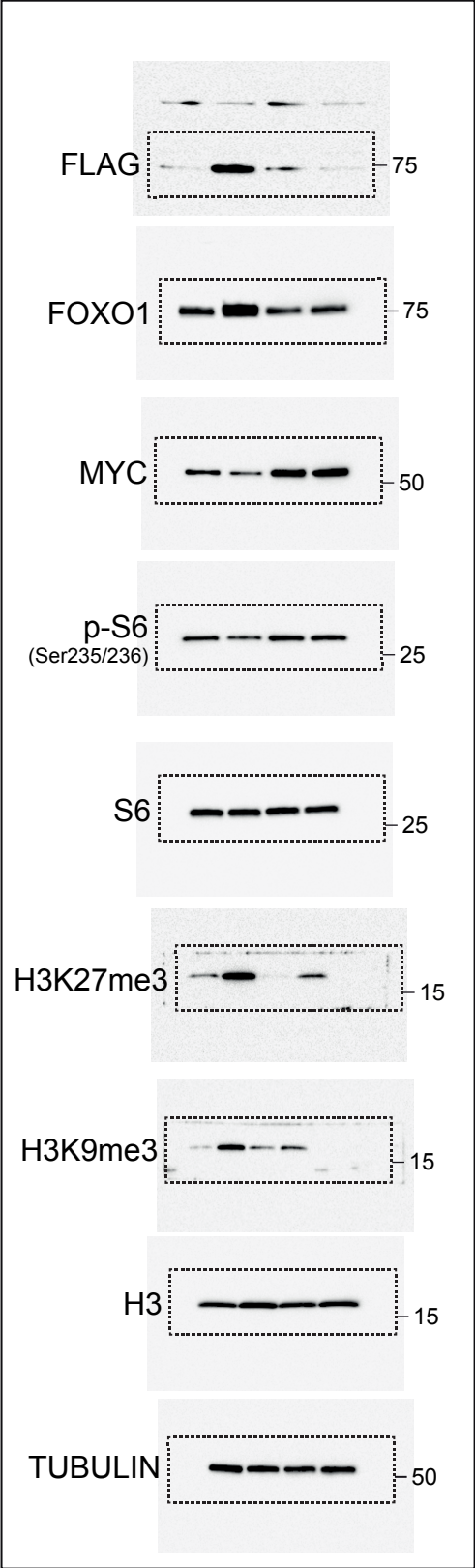

Supplement: Source Data Extended Data Fig. 1 — Unprocessed western blots. [file 41556_2021_637_MOESM19_ESM.pdf]

Source Data Extended Data Figure 2

Figure 2A

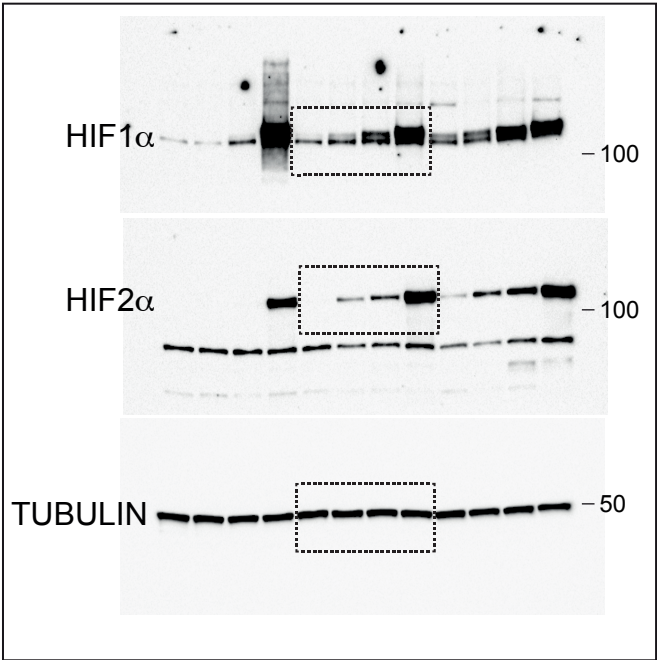

Figure 2D

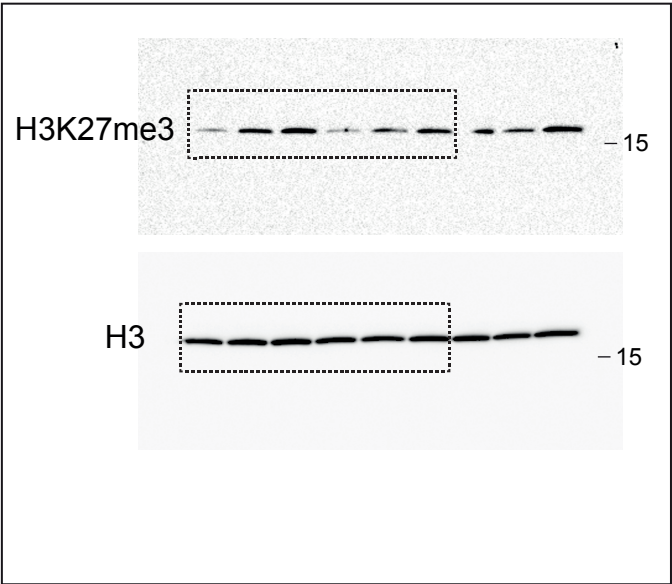

Supplement: Source Data Extended Data Fig. 2 — Unprocessed western blots. [file 41556_2021_637_MOESM20_ESM.pdf]

Figure 3F

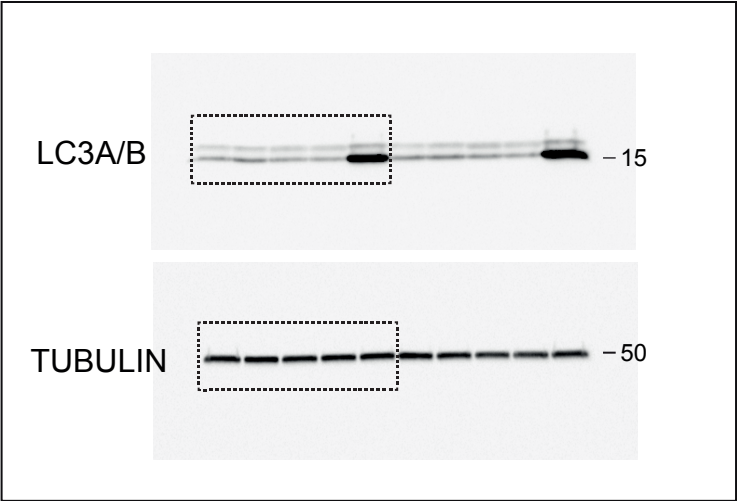

Supplement: Source Data Extended Data Fig. 3 — Unprocessed western blots. [file 41556_2021_637_MOESM22_ESM.pdf]

Figure 4D

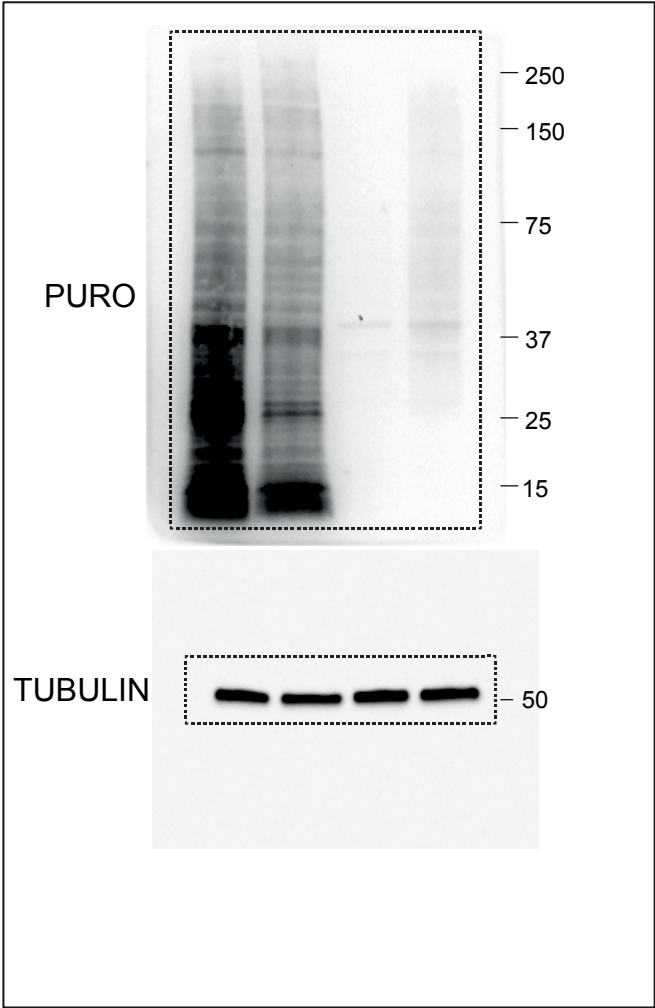

Supplement: Source Data Extended Data Fig. 4 — Unprocessed western blots. [file 41556_2021_637_MOESM23_ESM.pdf]

Source Data Extended Data Figure 7

Figure 7D

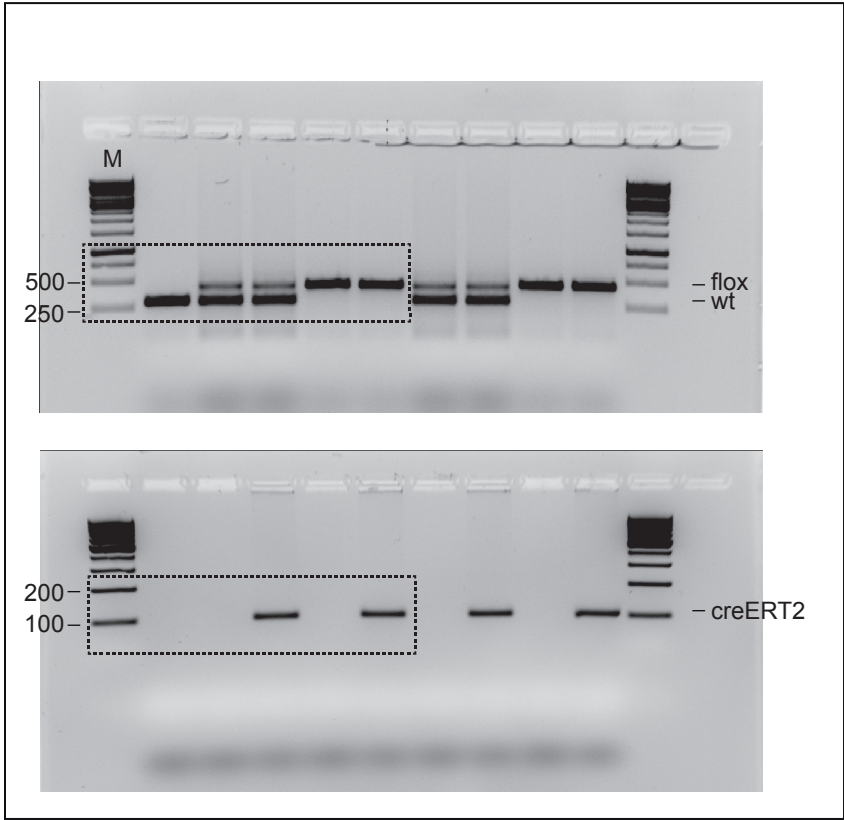

Figure 7E

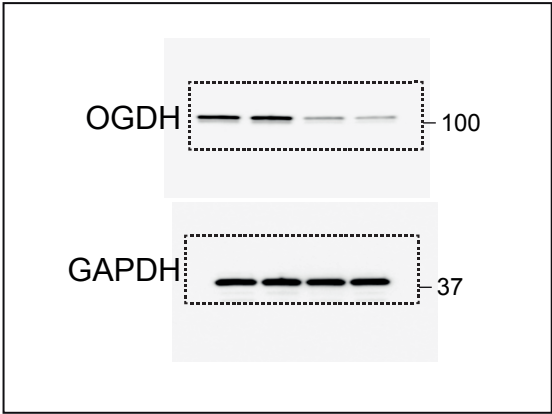

Supplement: Source Data Extended Data Fig. 7 — Unprocessed gels/blots. [file 41556_2021_637_MOESM27_ESM.pdf]

Source Data Extended Data Figure 8

Figure 8A

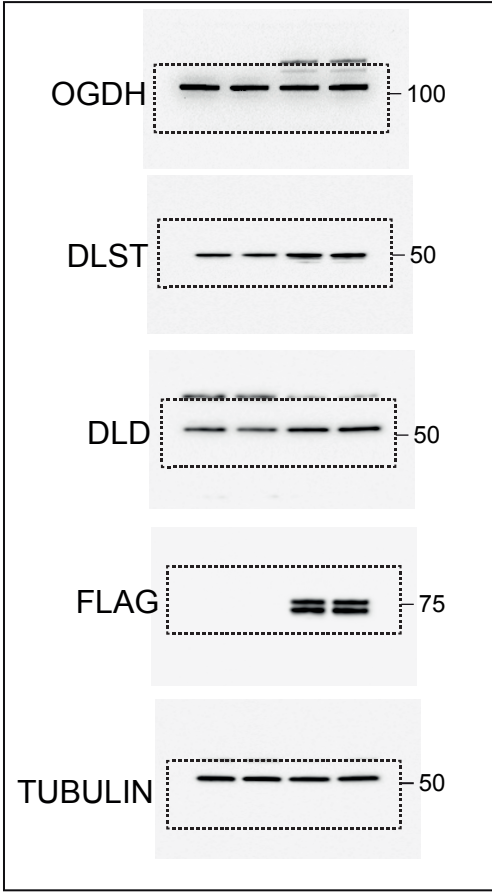

Figure 8B

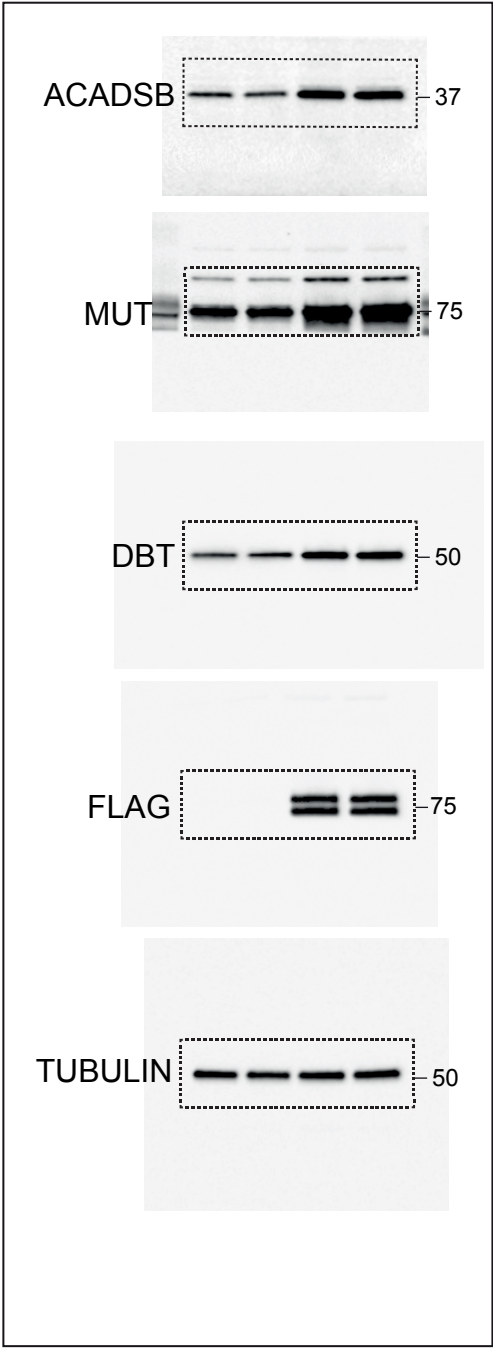

Figure 8C

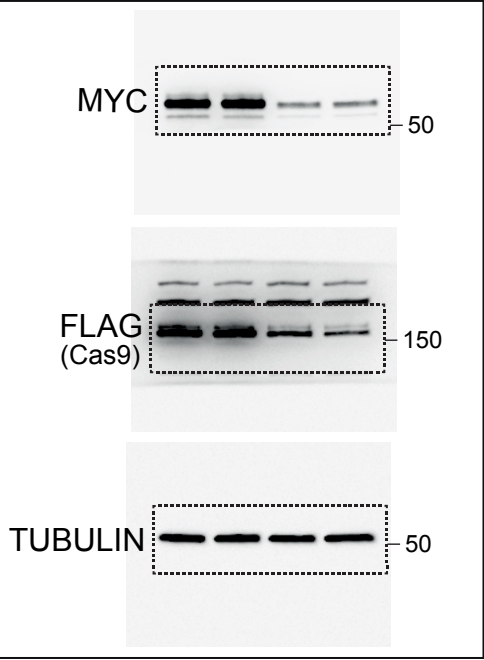

Supplement: Source Data Extended Data Fig. 8 — Unprocessed western blots. [file 41556_2021_637_MOESM29_ESM.pdf]

Figure 10A

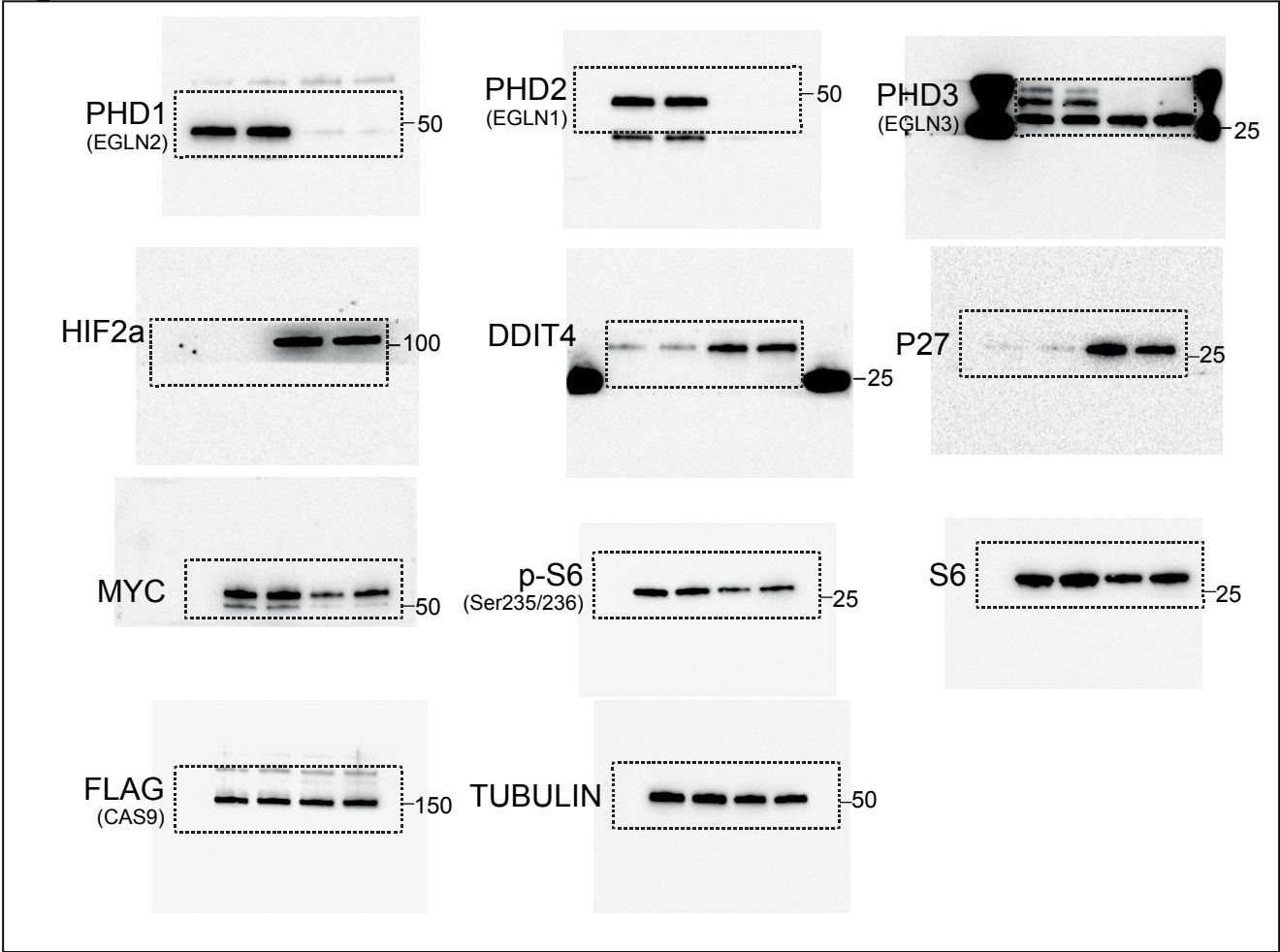

Supplement: Source Data Extended Data Fig. 10 — Unprocessed western blots. [file 41556_2021_637_MOESM31_ESM.pdf]
